# Accurate Ring Strain Energies of Unsaturated Three-Membered Heterocycles with One Group 13–16 Element

**DOI:** 10.1021/acs.inorgchem.2c00067

**Published:** 2022-04-20

**Authors:** Alicia Rey Planells, Arturo Espinosa Ferao

**Affiliations:** Departamento de Química Orgánica, Facultad de Química, Campus de Espinardo, Universidad de Murcia, 30100 Murcia, Spain

## Abstract

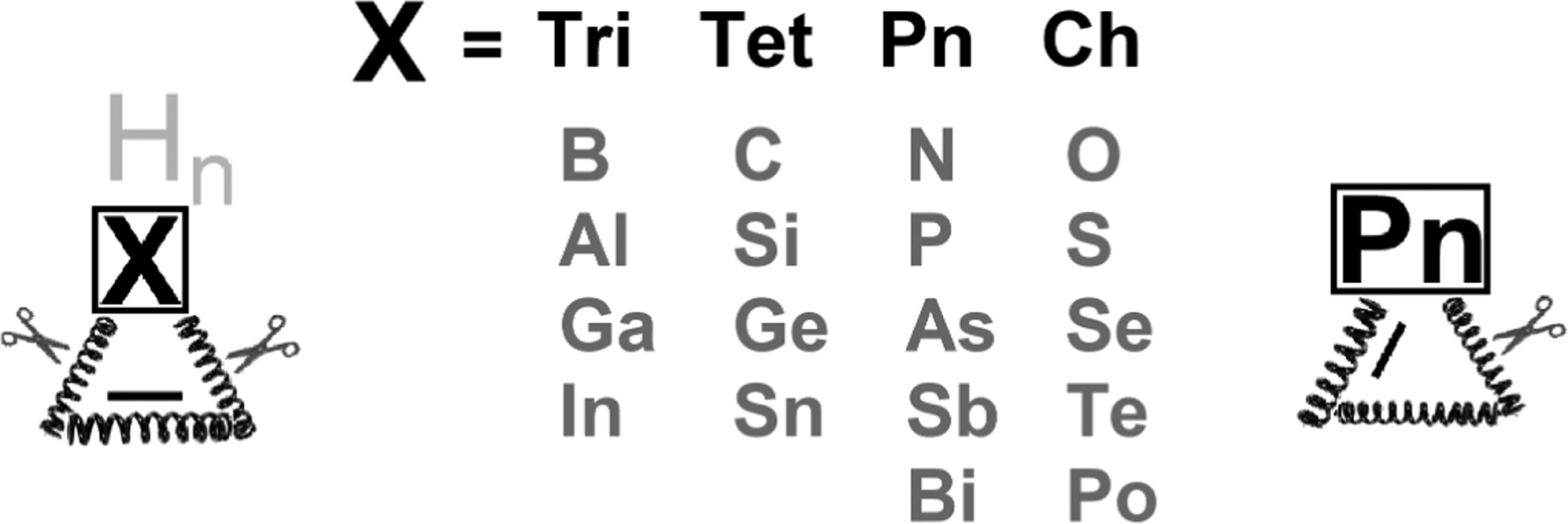

High-quality ring strain energy (RSE) data for 1*H-*unsaturated (CH)_2_X parent rings, where X is a group 13–16 element, are reported in addition to the 2*H*-isomers of the pnictogenirene rings. RSE data are obtained from appropriate homosdesmotic reactions and calculated at the DLPNO-CCSD(T)/def2-TZVPP//B3LYP-D3/def2-TZVP(ecp) level. 1*H-*Tallirene and 1*H-*plumbirene have unique donor–acceptor structures between an acetylene π(CC) orbital and an empty p orbital of a metallylene subunit (a Dewar–Chatt–Duncanson description) and therefore cannot be described as proper rings but as pseudocyclic structures. Also, 1*H-*indirene and 1*H-*oxirene lack ring critical points and constitute borderline cases of pseudorings. 1*H*-Unsaturated rings exhibit enhanced RSE compared to their saturated homologues. The mechanism of ring strain relaxation by increasing the s character in the lone pair (LP) of group 15–16 elements is remarkable and increases on descending the groups. Furthermore, RSE is affected by the aromatic character of group 13 rings and certain aromatic or antiaromatic character in group 14 or 15–16 rings, respectively, which tend to vanish on descending the group as shown by NICS(1) values. 2*H*-Unsaturated rings were found only for group 15 elements (although only 2*H*-azirine shows a proper cyclic structure) and displayed lower RSE (higher stability) than the corresponding 1*H*-isomers.

## Abstract 2

Accurate ring strain energies (RSE) are calculated for three-membered 1*H*-unsaturated rings containing a 13−16 group element and group 15 2*H*-isomers. The presence or absence of lone pairs at the heteroatom and the existence of intrinsic or residual (anti)aromaticity were shown to affect the RSE.

## Introduction

The chemistry of unsaturated three-membered heterocycles has been an important field of study for theoreticians and experimentalists for decades. Their peculiar geometrical characteristics lie in their inherent high ring strain and the presence of unsaturated bonds and heteroatoms, which introduce (de)stabilizing factors such as lone pair repulsion, σ-aromaticity, delocalization, and rehybridization.^1^ They are also a source of starting materials for the synthesis of a wide range of other more complex substances. For instance, the versatile cyclopropene is used as a reagent in the well-known Diels–Alder reactions^2^ or in its chiral version to produce enantiomerically enriched methylene- and alkylidenecyclopropane derivatives.^3^ Also, 2*H*-azirine is an important intermediate for the preparation of acyclic amino compounds^4^ and substituted aziridines,^5^ among other important reactions. And dimerization of borirene leads to 1,4-diboracyclohexadiene.^6^

In the study of three-membered unsaturated heterocycles containing (at least) a group 13–16 heteroatom, the essential concept of aromaticity (or antiaromaticity in case) in chemistry comes into play.^7^ Numerous studies are reported in the literature that try to elucidate how this condition affects the geometrical and electronic aspects of this type of rings. Although the combination of acetylene and silylene was suggested to be an appropriate way of describing silacyclopropane (silirane), the stability exhibited when successfully synthesized by Seyferth in 1972^8^ hinted at considering that its unsaturated version, silacyclopropene (1*H-*silirene), might also be stable due to some predicted aromatic character,^9^ as a few years later demonstrated by the synthesis of the tetramethyl-1-silacycloprop-2-ene derivative by Conlin et al.^10^ The heavier analogues 1*H*-germirenes were reported to be stable species,^11^ and a high ring strain energy (RSE) of 44.4 and 47.6 kcal/mol were computed for the parent and 1-bromo-substituted rings,^12^ respectively, whereas a somewhat lower strain was obtained for the corresponding 1*H*-silirene (RSE = 40.6 and 42.0 kcal/mol, respectively). On the other hand, the rather unstable 1*H-*thiirene was the first formally antiaromatic (4π electron) heterocycle prepared,^13^ which tries to alleviate this destabilization by lengthening the C–S bonds and shortening the C=C bond. No less interesting is 1*H-*oxirene, which has given rise to considerable controversy as to whether the *C*_2*v*_-symmetric structure exists as a local minimum or a saddle point energy between two degenerated low-symmetry (*C*_*s*_) minima, which greatly turned out to depend on the computational level used.^14^ However, it seems unanimous that its derivative dimethyloxirene does constitute a high-symmetry (*C*_2*v*_) energy minimum.^15^ As for the antiaromatic pnictogens group, the three-membered N- and P-containing unsaturated rings constitute exciting fields in heterocyclic chemistry. The chemistry of aziridines and azirines has been a tremendously exploited field since the review of Padwa and Woolhouse.^16^ Two types of azirines can be formed, namely, 1*H*-azirine and 2*H*-azirine, both exhibiting unique reactivity such as the ability to act as either nucleophile or electrophile,^17^ as well as the possibility of being used as precursors to more complex N-containing molecules.^4^ Their structures have been well studied recently.^18^ A 2*H*-azirine is the highly strained key intermediate in the Neber rearrangement that transforms ketoximes into α-amino-ketones.^19^ In contrast, the development of the chemistry of three-membered P-containing rings was somewhat delayed, starting with Wagner’s discovery of phosphirane in 1963.^20^ A few years later, 1*H*-phosphirene and 2*H*-phosphirene were discovered by Mathey^21^ and Regitz,^22^ respectively, both fields growing rapidly to become key building blocks in organophosphorus chemistry.^23^ But it is not all about antiaromaticity: in 1998, the remarkable stability of 2π-electron aromatic 1*H-*borirene was reported to be the most stable isomer of BC_2_H_3_, in contrast to its aluminum counterpart, which highlights the ability of the B atom to form stable rings.^24^

**Figure 1:**
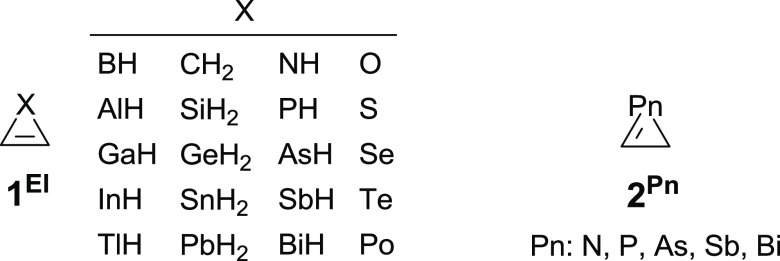
1*H*- (**1**) and 2*H*-unsaturated (**2**) three-membered heterocycles studied.

In the present work, high-quality benchmark RSE (ring strain energy) data are reported for parent (CH)_2_X rings, where X is a group 13–16 element (Figure 1), with its most characteristic covalency (3, 4, 3, and 2 for groups 13–16, respectively) completed by bonds with H, and is designated as **1**^**El**^ (“**El**” refers to the heavy element of group X). In addition, a discussion of possible factors affecting the ring strain as well as (anti)aromaticity in each case is presented. The 2*H*-isomers **2** were also studied, although only those with pnictogen atoms were found as stable species.

## Results and Discussion

### 1*H-*Unsaturated Ring Structures, **1**

The main geometrical parameters for all computed (see the Experimental Section) energy minima of rings **1**^**El**^ (Figure 1) were collected (Table 1) to obtain an appropriate description of their structures.

**Table 1:** Calculated (B3LYP-D3/def2-TZVPecp) C=C Bond Distances (Å) and C–X–C Bond Angles (Degrees, in Parentheses) for Compounds **1**^**El**^

group 13	group 14	group 15	group 16
**B** 1.346 (54.6)	**C** 1.287 (50.6)	**N** 1.269 (49.5)	**O** 1.269 (48.0)
**Al** 1.369 (43.3)	**Si** 1.330 (43.1)	**P** 1.292 (41.1)	**S** 1.271 (40.1)
**Ga** 1.356 (41.8)	**Ge** 1.319 (40.1)	**As** 1.287 (37.8)	**Se** 1.268 (36.8)
**In** 1.330 (36.1)	**Sn** 1.320 (36.3)	**Sb** 1.287 (34.3)	**Te** 1.267 (33.4)
**Tl** 1.201 (22.2)	**Pb** 1.203 (23.7)	**Bi** 1.280 (32.4)	**Po** 1.261 (31.5)

**Figure 2:**
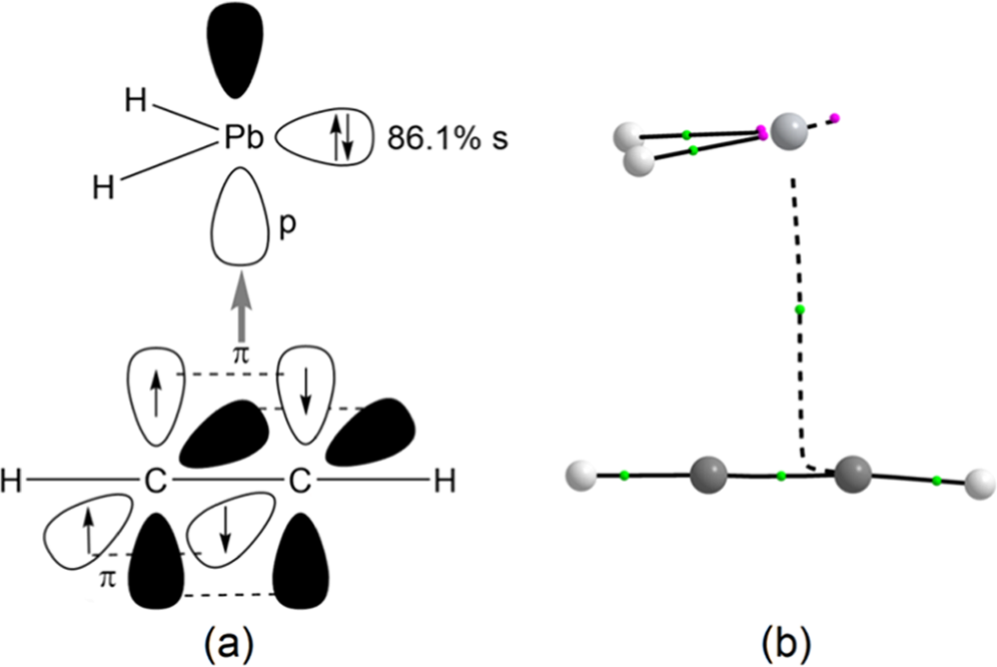
Sketched representation of DCD interaction (a) and computed (B3LYP/def2-TZVPPecp) structure with BCP (small green spheres), nuclear nonattractive critical points (NNACP, small pink spheres), and bond paths (b) for pseudo-plumbirene (**1**^Pb*^).

The C=C bond distance maintains a general tendency to decrease on moving right within the row, probably due to the increasing electronegativity of the heteroatom, as also observed for the fully saturated counterparts and tested on simple acyclic X–CH_2_–CH_2_–X model species (X = BH_2_, CH_3_, NH_2_, OH).^25^ On descending within the same group, the C=C bond distance decreases regularly, reaching limiting values (also very acute C–X–C bond angles) for rings containing heteroatoms with the highest metallic character Tl and Pb. Thus, **1**^Tl*^ (1*H*-thallirene) and **1**^Pb*^ (1*H*-plumbirene) present a pseudocyclic structure (designated with the asterisk) according to the Dewar–Chatt–Duncanson (DCD) model,^26^ where the HCCH moiety displays a high triple-bond (acetylene) character (WBI = 2.936 and 2.868 for **1**^Tl*^ and **1**^Pb*^, respectively). Hence, these pseudorings are better described by a dative bonding from a filled π(C≡C) orbital in an acetylene subunit to an empty p atomic orbital (AO) in the X (TlH or PbH_2_) subunit (Figure 2). The energies corresponding to this interaction, obtained from the second-order perturbation theory (SOPT) of the Fock matrix in the natural bond orbital (NBO) basis, for pseudo-thallirene and -plumbirene are 6.28 and 22.84 kcal/mol, respectively. Atoms-in-molecules (AIM) analysis^27^ also supports this description by lacking a ring critical point (RCP) and attributing a single bond critical point (BCP) between the organic and metallic fragments (see Figures 2 and S1). This different behavior exhibited by **1**^Tl*^ and **1**^Pb*^ could be attributed to the well-known inert pair effect, typical for this type of post-transition-metal elements, which affects the tendency of the pair of the outermost s electrons not to be shared, making more favorable a structure with DCD-type interaction between two subunits.

1*H*-Indirene (**1**^In*^) represents a borderline case as it does not show a typical DCD structure due to the double C–C bond character (Table 1) (WBI = 2.028; natural localized molecular orbitals NLMO-BO = 2.007), but it can be categorized as a pseudocycle as it lacks RCP, only displaying one BCP at the shorter C–In bond (2.063 Å) and being the other slightly elongated (2.210 Å) (see the Supporting Information).

**Figure 3:**
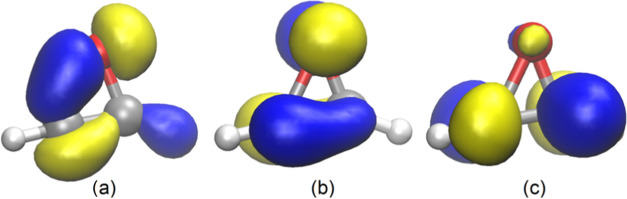
Computed (B3LYP-D3/def2-TZVP) Kohn–Sham isosurfaces (0.07 au) for (a) HOMO – 1, (b) HOMO, and (c) LUMO of pseudo-oxirene (**1**^O*^).

On the other hand, for the controversial oxirene **1**^O*^ geometry (*vide supra*), it was found that at the MP2(FU)/6-31G(d) level or using GGA functionals (BLYP, BP91, PBE, HCTH, HCTH147, and HCTH407), with various double- and triple-ζ quality basis sets with a variety of extra polarization functions, the *C*_2*v*_-symmetric **1**^**O**^ constitutes a transition state (TS) between two degenerate low-symmetry (*C*_*s*_) minima, whereas at the HF/6-31G(d) level, *C*_2*v*_-**1**^**O**^ is a true minimum; for hybrid functionals (B3LYP, B3P91, PBE0, B97, B97-1, and B3P86), a disparity of results was obtained.^28^ We have obtained similar results with the PBEh-3c^29^ functional to those with HF calculations. At the working level of theory used for optimizations throughout the current study (see the Computational Details section), oxirene **1**^O*^ has a *C*_*s*_ symmetry distorted structure with long (C_1_–O 1.673 Å) and short (C_2_–O 1.376 Å) bonds, interconverting through a very low-lying *C*_2*v*_-symmetric TS (ΔΔ*E*_ZPE_^‡^ = 0.28 kcal/mol). The weak and long C_1_–O bond is due to the fact that it is formed involving two almost pure p AO at carbon (94.3% p) and oxygen (96.6% p), as shown in the highest occupied molecular orbital (HOMO) – 1 isosurface, with HOMO and lowest unoccupied molecular orbital (LUMO) displaying π*(C–O) and π*(C–C) character, respectively (Figure 3). However, oxirene lacks a ring critical point (RCP) therefore being categorized as a pseudocyclic structure.

### Ring Strain Energy Values and Related Parameters for Compounds **1**

**Scheme 1:**
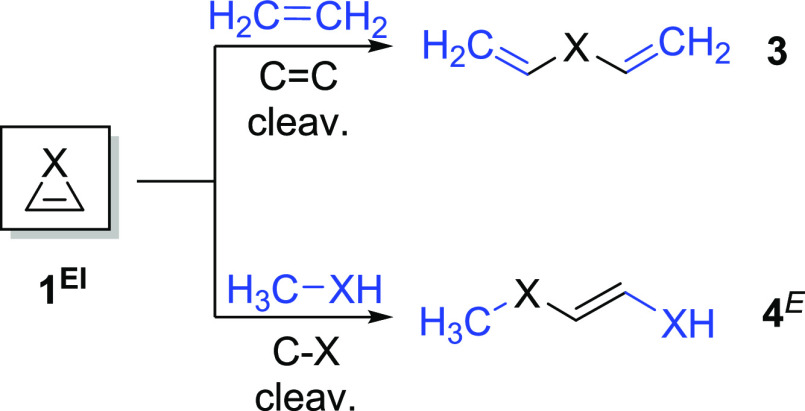
Homodesmotic (RC4) Ring-Opening Reactions Used for the Estimation of RSE for **1**^**El**^

Ring strain is one of the most remarkable aspects of small rings as it is closely linked to chemical reactivity and has been found to affect properties such as the stereochemical stability of σ^3^λ^3^-pnictogen ring atoms.^30^ It frames the driving force for a ring to be transformed into open-chain products.^31^ RSEs were calculated using homodesmotic reactions (reaction class 4 or “RC4”), which constitute the penultimate type in a hierarchy of increasingly accurate processes, due to the conservation of larger fragments, according to a recent classification and redefinition of reaction types used in thermochemistry.^32^ Good estimates for RSE concerning saturated^25,31,33^ and unsaturated^12,34^ three-membered heterocycles were obtained according to this type of thermochemical reaction schemes. The highest ranked hyperhomodesmotic reactions (reaction class 5 or “RC5”) were skipped because they have been shown to give rise to very similar RSE data to RC4,^25^ but being somewhat problematic in some cases by introducing undesired noncompensated interactions due to the presence of longer chains. Therefore, the RSE for unsaturated three-membered (CH)_2_X heterocycles with group 13–16 heteroelements **1**^**El**^ was calculated using the RC4 scheme (Scheme 1) and obtained as the average of the opposite energy (including the zero-point energy correction) for the three bond cleavage reactions corresponding to the three endocyclic bond-breaking reactions (one C=C and two C–X bonds) (Table 2). Although in principle, the *Z*-isomer of the homodesmotic C–X bond cleavage product should be the most suitable diastereomer according to its similarity with the endocyclic C=C bond, it introduces undesirable long-distance steric clashes, and hence only the *E*-isomer was used. The introduction of a C=C double bond in a small 3MR is expected to drastically increase the ring strain owing to the higher energy that should be required to compress an acyclic C=C–El bond angle from *ca.* 120° to the *ca.* 60° expected in the three-membered cyclic species, compared to the shorter bond angle compression required in a saturated C–C–E moiety. This is long time known since the first report of RSE for cyclopropene (53.2 kcal/mol)^35^^a^ in 1976, compared to that of cyclopropane (27.9 kcal/mol).^25^

**Table 2:** RC4-Derived Calculated (DLPNO-CCSD(T)/def2TZVPPecp) RSE Values (kcal/mol) for Compounds **1**^**El**^ and **2**^**Pn**^

**1^El^**				**2**^**Pn**^
group 13	group 14	group 15	group 16	group 15
**B** 31.15 (68.8^36^^c^)	**C** 53.97 (53.2,^35^^a^ 55.0,^35^^b^ 55.5,^35^^c^ 54.1,^35^^d^ 55.7,^35^^e^ 54.1,^35^^f^ 54.1,^35^^g^ 56.0^37^)	**N** 71.02 (77.3^37^)	**O**^b^ 76.10 [71.31]^a^ (74.9,^35^^f^ 82.4^37^)	**N** 41.19 (43.9,^37^ 41.9^38^)
**Al** 42.09	**Si** 41.94 (45.5,^35^^b^ 34.5,^35^^c^^c^ 49.0^39^)	**P** 38.03 (39.0^37^)	**S** 51.51	**P**^b^ 32.57 [31.38]^a^ (34.3^37^)
**Ga** 45.46	**Ge** 46.62 (44.4^12^)	**As** 35.89	**Se** 45.25	**As**^b^ 32.73 [32.95]^a^
**In**^b^ 46.22 [46.29]^a^	**Sn** 45.01	**Sb** 30.95	**Te** 38.15	**Sb**^b^ 30.91 [29.71]^a^
		**Bi** 26.92	**Po** 33.17	**Bi**^b^ 28.12 [28.11]^a^

a Values calculated at the CASSCF(*n*,6)/MRACPF/def2-SVPD level are presented in square brackets (see the Supporting Information for the specific number of active orbitals used, *n*).

b Pseudocyclic structures.

c See text.

Data collected in Table 2 are in reasonable agreement with previously reported RSE data that were obtained using different computational levels and, in most cases, lower-quality thermochemical equations (*e.g.*, isodesmic reactions). Worth is mentioning the erroneously reported value of 34.5 kcal/mol for the RSE of **1**^**Si**^, which was referenced to a publication where this was not mentioned.^35^^c^ On the other hand, the extremely high RSE value reported for the 1*H*-borirene **1**^**B**^ ring is based on the wrong assumption that an increase from the saturated counterpart borirane (reported RSE = 43.7 kcal/mol) could be estimated from the RSE difference (Δ_RSE_ = 24.8 kcal/mol) between cyclopropene (**1**^**C**^) and cyclopropane.^36^

For those (pseudo)rings with a diagnostic T1, a parameter widely used to evaluate the multi- or single-reference character, close to 0.02 (no ring or pseudo-ring exceeds this value). the multireference RSE value has been calculated by selecting the appropriate number of *n* electrons and *m* active orbitals (Table S3) to analyze possible differences between the two methods. The lowest values of single-reference contribution are shown for 1*H*-unsaturated **1**^In*^ (76.2%) and **1**^O*^ (80.6%) both with the highest diagnostic value T1 (0.02), although the RSE values for the two methods are quite similar for **1**^In*^ (despite having considerable multireference character). The pseudo-ring **1**^O*^ has a marked multireference character (of almost 20%), and this is reflected in a difference of about 5 kcal/mol between the two calculation methods.

**Figure 4:**
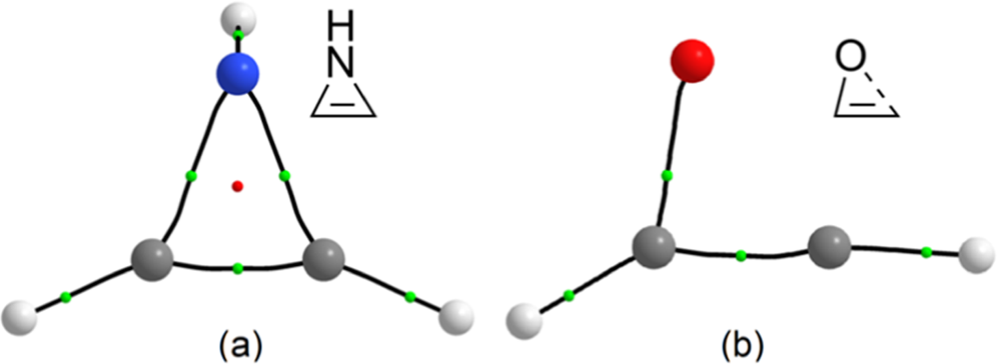
Computed (B3LYP/def2-TZVPP) BCP (small green spheres), RCP (small red sphere), and bond paths for (a) **1**^**N**^ and (b) **1**^O*^.

As described in the previous section, the pseudocyclic oxirene ring **1**^O*^ lacks an RCP and exhibits an extremely high RSE value (that should be taken with caution), paralleling the high exothermicity of its low barrier (Δ*E*_ZPE_^‡^ = 3.63 kcal/mol) isomerization to ketene (Δ*E*_ZPE_ = −77.57 kcal/mol; reported: −80.02 kcal/mol^40^). Despite the similar high RSE value of 1*H*-azirine, **1**^**N**^, its proper cyclic structure is supported by the existence of BCPs for every endocyclic bond (see Table S1), as well as an RCP (Figure 4) with a high value of Lagrange kinetic energy density per electron *G*(*r*)/ρ(*r*) (see Table S1), which have been shown to correlate with RSE.^41^ Moreover, calculation of the relaxed force constant,^42^ a numerically stable and fully transferable parameter related to the stiffness of bond lengths or bond angles, reveals that the C–C–El bond angle of pseudocyclic **1**^O*^ (*k*^0^ = 0.124 mdyn·Å) is below one-tenth of that for **1**^**N**^ (*k*^0^ = 1.572 mdyn·Å), which also points to an inherent difference between pseudocyclic and cyclic characters, respectively.

**Figure 5:**
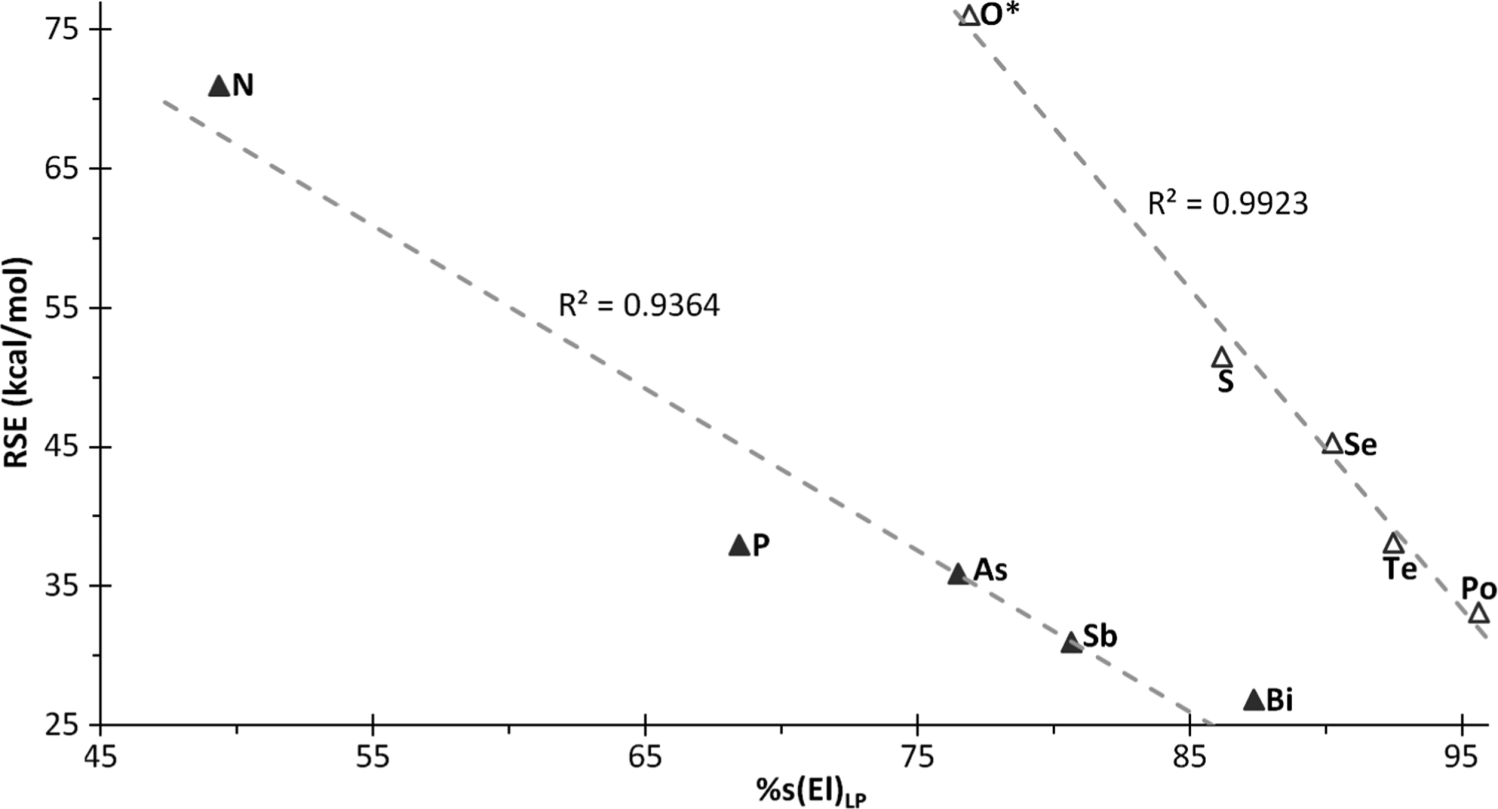
Plot of RSE *vs* s character (%) of AO used by pnictogen and chalcogen heteroatoms “El” lone pairs (LPs) in **1**.

Unsaturated rings with heteroatoms belonging to groups 15 and 16 experience the lone pair (LP) s character-enhancing effect on the RSE (Figure 5),^43^ in line with the observed behavior of the already reported homologous saturated rings.^25^ This increase of the LP s character causes the increase in p character for the AOs used by heteroatom “El” in the endocyclic C–El bonds, resulting in an sp*^n^*-type hybridization with *n* > 3 (*i.e.*, beyond 75%p). As the s character of the LP AO increases on descending the group, the RSE for the corresponding heterocycle **1**^**El**^ decreases (Figure 5). The alternative plot of RSE against the p character of the AO used by the heteroatom “El” for its endocyclic C–El bonds, %p(El)_C–El_, shows a slightly worse correlation for groups 15 and 16 (*R*^2^ = 0.8692 and 0.9826, respectively) (see Figure S4), while for groups 13 and 14, the expected almost constant p character (around 72 and 81%, respectively) is observed due to the absence of LPs.

Also, the relaxed force constants *k*^0^ computed for C–El bonds in heterocycles with heaviest group 13–16 elements **1**^**El**^ parallels the behavior of RSE, as *k*^0^_C–El_ decreases (bond elasticity increases) with the increase of %p(El)_C–El_ (Figure S2). The lighter elements (except C) fall out of the correlation, exhibiting unexpectedly low (for N and O) or high (for B) *k*^0^_C–El_ values. For groups 13 and 14, the C–El bond stiffness is higher than for groups 15 and 16, and its decrease on moving down the group is much more pronounced. In the case of **1**^O*^ and **1**^In*^, both %p(El)_C–El_ and *k*^0^_C–EI_ are averaged values for the two different C–El bonds in the pseudo-ring. Furthermore, due to the orthogonality of p AOs, an increase of p character should move the electron density of the σ(C–El) bond out of the hypothetical C–El straight bond line. Indeed, bond bending from NHO (natural hybrid orbital) directionality analysis shows a remarkable correlation between the deviation angle and the %p(El)_C–El_, only for group 15 and 16 elements (Figure S3). The angular deviation increases on moving down the group, being more pronounced (higher slope) for chalcogens than for pnictogens. This is indeed the opposite trend observed for RSE *versus* %p(El)_C–El_ (Figure S4), which suggests the easiness of bond bending as a mechanism for relieving the ring strain. For groups 13 and 14, the %p(El)_C–El_ values vary within a small range (*ca.* 70–74 and 79–82%, respectively) and, again, the angular deviation increases on descending the group (except for pseudocyclic **1**^In*^).

However, despite the strain-relieving effect of the LP s character in group 15–16 unsaturated heterocycles **1**^**El**^, the high RSE values displayed compared to their saturated counterparts (which represent the least strained rings within the series), and especially for **1**^**N**^ and **1**^**O**^ (also for **1**^**S**^ and **1**^**Se**^), are due to their intrinsic antiaromatic character or the geometric deformation to partially mitigate this electronic destabilization. In fact, the observed elongation of the endocyclic C–El bonds (Table S1) compared to analogous C–El bonds in acyclic species (taking the homodesmotic ring-opening products as a reference; see Table S2) is more pronounced for **1**^**N**^ (9.7%) and **1**^**O**^ (11.6% in average) and decreases significantly from the third period onwards (**1**^**P**^ 0.9%; **1**^**As**^ 1.2%; **1**^**Sb**^ 1.0; **1**^**Bi**^ 1.4%; **1**^**S**^ 5.8; **1**^**Se**^ 5.3; **1**^**Te**^ 4.6%; **1**^**Po**^ 4.6%), in accordance with the decreased antiaromaticity.

**Figure 6:**
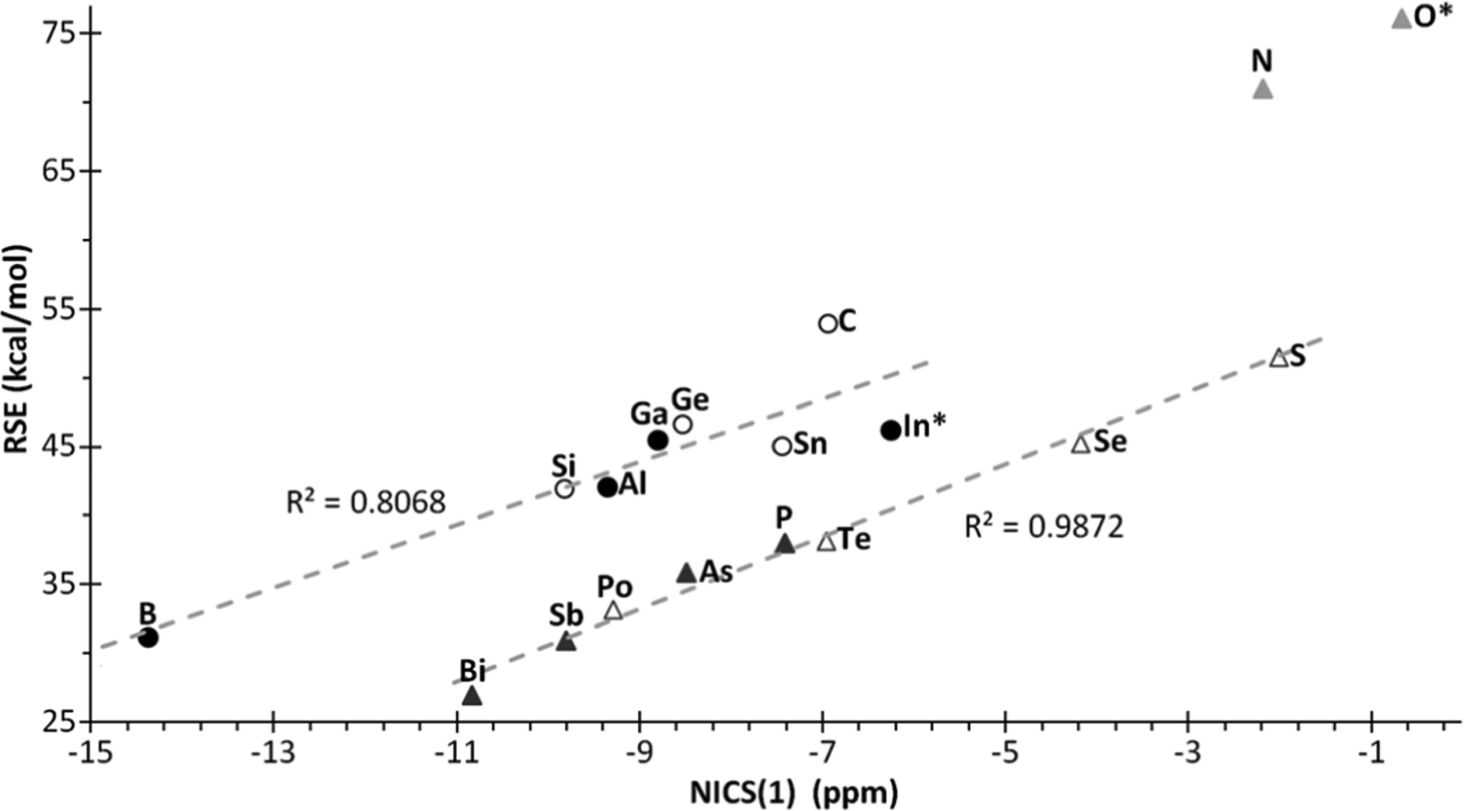
Plot of RSE *vs* computed (B3LYP/6311+G(d,p) or def2-TZVPP) NICS(1) (ppm) for compounds **1**. Excluding **1**^**N**^ and **1**^O*^ from the linear correlation.

Although (anti)aromaticity is not an observable, several parameters have been described trying to quantify it from the geometrical, energetic, and magnetic perspectives.^44^ One of the most widely used parameters is the nucleus-independent chemical shift (NICS)^45^ which allows the aromatic character to be quantified so that the more negative the NICS value, the higher the aromatic character. As isotropic NICS values calculated at the ring centroid are strongly influenced by the σ-contributions,^46^ NICS(1) (computed at 1 Å above and below the ring centroid) are usually employed.^47^ The NICS(1) values for **1**^**El**^ were calculated at the standard B3LYP/6311+G(d,p) level^47^ for elements of the second to fourth rows and using the def2-TZVPP basis set for elements of the fifth and sixth rows. At first sight, a clear correlation between NICS(1) and RSE is observed (Figure 6), with almost linear character for elements having (groups 15–16) or not having LPs (groups 13–14), if **1**^**N**^ and **1**^O*^ are excluded. As expected, the most antiaromatic rings are those with the lightest group 15–16 elements (nitrogen and oxygen) due to the most efficient overlap of the heteroelements LP with the π(C=C) orbital. Hence, antiaromaticity considerably decreases on moving down in groups 15 and 16 and varying almost linearly with RSE. As foreseeable, **1**^**B**^ is the most aromatic ring of all herein described,^48^ therefore showing the most negative NICS(1) value and entailing a remarkable decrease in RSE (Figure 6). Again, on descending group 13, the heavier congeners of Al, Ga, and In use an empty p AO with increasing principal quantum number, which decreases the efficiency of the overlap with the C=C π-system, hence decreasing the aromaticity of the corresponding unsaturated heterocycles **1**^**El**^ and increasing their RSE.

**Figure 7:**
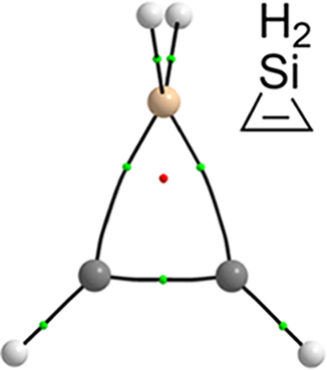
Computed (B3LYP/def2-TZVPP) BCP (small green spheres), RCP (small red sphere), and bond paths for **1**^**Si**^.

As for group 14, it is worth mentioning the remarkable aromatic character, according to its highly negative NICS(1) value, observed for the **1**^**Si**^ ring (Figure 6), which has been explained in terms of using empty low-lying 3d*_xz_* orbitals of silicon playing a similar role to the positive vacancy (2p*_z_*) in the C atom of cyclopropenyl cation.^10^ It should also be ascertained that the proposed^10^ DCD-type structure for the 1*H*-silirene ring **1**^**Si**^, although suggested by the HOMO isosurface (Figure S5), must be ruled out, not only on the basis of its geometric features (Table 1) but also supported by the atoms-in-molecules (AIM) analysis,^27^ showing three BCPs for the three endocyclic bonds and an RCP (Figure 7), and not a single BCP between the hypothetical acetylene and silylene moieties, as is would be expected for a DCD-type structure. The aforementioned stabilization by d-orbital participation is supported by the NBO analysis, indicating *E*_SOPT_ = 3.88 kcal/mol associated with an electron transfer from a filled π(C=C) to an almost empty Si orbital with almost pure (95.9%) *d*-character. This SOPT energy in **1**^**Ge**^ (1.24 kcal/mol, 90.7% *d*-character) and **1**^**Sn**^ rings (1.10 kcal/mol, 69.5% *d*-character) decrease due to participation of 4d and 5d orbitals, respectively, with increasing energy gap, whereas **1**^**C**^ lacks d orbitals, which justifies its nonaromatic character. However, the origin of the diatropic ring current (aromaticity) accounting for the observed highly negative NICS(1) value for **1**^**Si**^ can mostly arise from an energetically more important sacrificial hyperconjugation effect between the π(C=C) and the two σ*(Si–H) molecular orbitals, each amounting to *E*_SOPT_ = 10.64 kcal/mol. The energy of the analogous double SOPT interaction decreases for the **1**^**Ge**^ and **1**^**Sn**^ rings (8.24 and 5.22 kcal/mol respectively), in line with their lower aromaticity. Similarly, for groups 15 and 16, whereas **1**^**N**^ and **1**^O*^ lack d-orbitals, a small yet significant electron transfer is observed for **1**^**P**^ and **1**^**S**^ from π(C=C) to empty d-orbitals (82.3 and 60.4% *d*-character, respectively), the SOPT interaction amounting to 5.89 and 5.74 kcal/mol, respectively. This suggests a certain (small) aromatic component, which has an effect on a decrease of RSE and a more negative NICS(1) resulting from a small diatropic current. This effect decreases (lower *E*_SOPT_) for heavier elements due to the increased energy gap between the donor π(C=C) orbital and the acceptor El-centered empty AO with increasingly lower *d*-character.^49^ The reason for the increase in the aromaticity-related NICS(1) descriptor as one moves down in groups 15 and 16 is not trivial but could be tentatively explained on the basis of the increasing size of the heavier atoms, whose inner-shell electrons could give rise to heteroatom-centered diatropic currents that could interfere in the overall NICS(1) value. The use of NICS(2) values (computed 2 Å away from the ring centroid) did not provide a clearer picture, and the same trends were observed when plotted against RSE (see Figure S6), with good linear correlations within groups and all rings displaying lower negative values than benzene.^47^ For group 15 rings, a decrease of sacrificial hyperconjugation of the π electron density with the σ*(El–H) molecular orbital on descending the group could also explain the decrease in aromaticity (*E*_SOPT_ = 8.54, 6.40, 4.61, and 3.59 kcal/mol for **1**^**P**^, **1**^**As**^, **1**^**Sb**^, and **1**^**Bi**^, respectively).

Nevertheless, as a consequence of the very high energy of the d-orbitals from the third row onward, the above-described effect of aromatic components could be negligible in this type of rings^50^ and, instead, other substituent-depending hyperconjugation effects may come into play.

**Figure 8:**
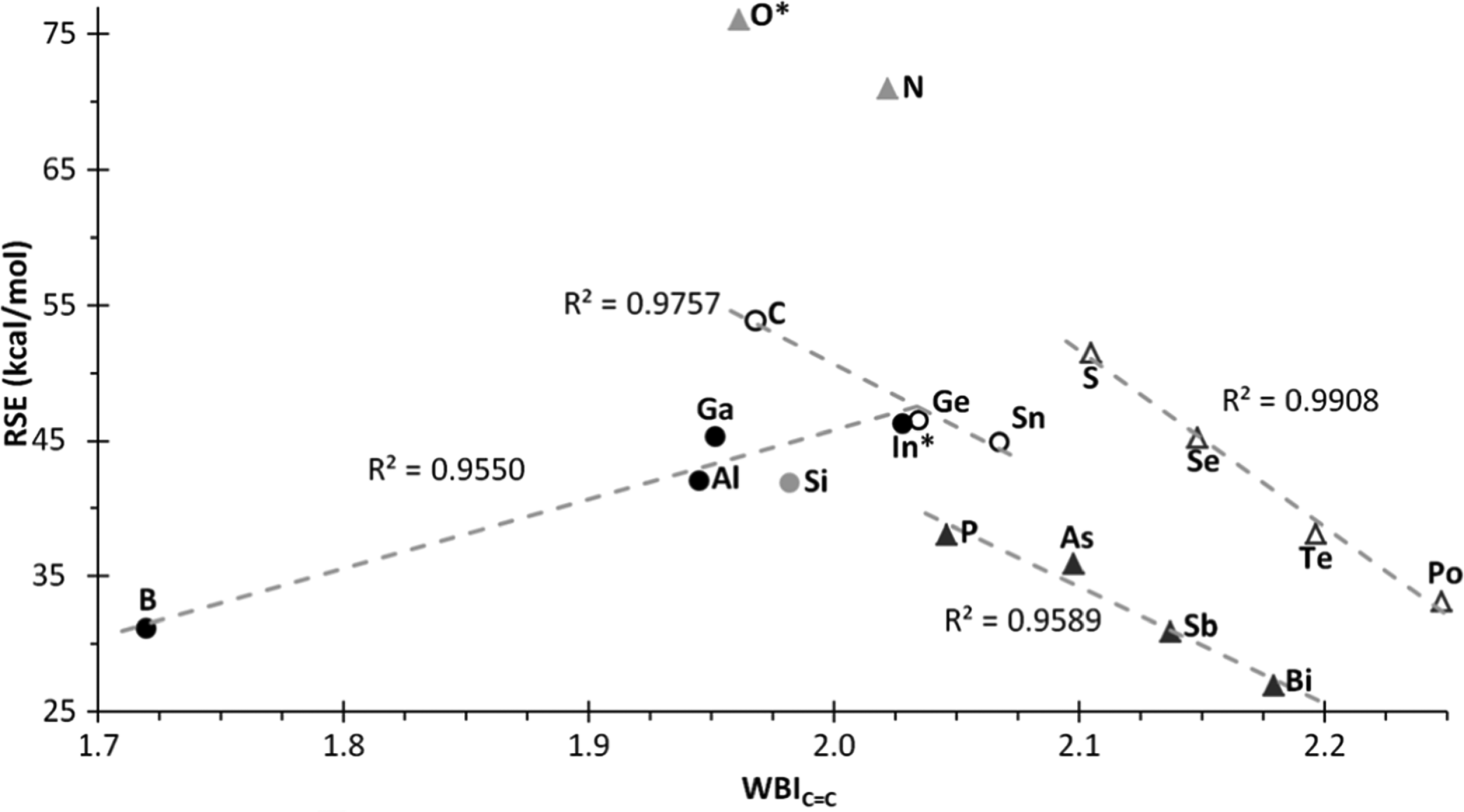
Plot of RSE *vs* WBI_C=C_ for compounds **1**, excluding **1**^**Si**^, **1**^**N**^, and **1**^O*^ from the linear correlation.

RSE may also be related to the strength of endocyclic bonds. Among bond-strength-related parameters, the widely used Wiberg bond index (WBI)^51^ as well as the bond order provided by the Natural Localized Molecular Orbitals (NLMO)^52^ analysis (NLMO-BO), less prone to overestimate the covalent character of the bond,^53^ were computed for all **1**^**El**^ heterocycles. The representation of the RSE *versus* both WBI (Figure 8) and NLMO-BO (see Figure S7) of the C=C bond show that for groups 14–16, as the ring strain decreases when moving down the group, the C=C bond order increases, but without reaching a value close to a genuine triple-bond character as in the case of DCD-type pseudorings (WBI/NLMO-BO = 2.934/2.943 and 2.867/2.902 for **1**^Tl*^ and **1**^Pb*^, respectively). For **1**^In*^, the WBI for the C=C bond (2.028) is clearly overestimated compared to the NLMO-BO (1.885), the latter falling out of the linear correlation of its group (Figure S7) probably because of its pseudocyclic character. 1*H*-Silirene **1**^**Si**^ is also markedly outside the linear correlation of group 14 (for both WBI and NLMO-BO), most likely due to its exceptional aromaticity with respect to the rest of the tetrel-based unsaturated rings, leading to a decrease in the RSE but without greatly affecting the strength of the C=C moiety. The most aromatic ring, 1*H*-borirene (**1**^**B**^), shows the lowest C=C bond orders (WBI = 1.720; NLMO-BO = 1.873) owing to the effective conjugation of the vacant p orbital in B to the C=C bond, drastically suppressed in the less aromatic and more strained **1**^**Al**^ and **1**^**Ga**^ rings (Figure 8).

In all, despite the expected large difference in RSE between 1*H*-unsaturated 3MRs and their saturated analogues, this turned out to be true only for **1**^**C**^. For the heaviest group 14 elements, this difference becomes less pronounced because of the aromatic stabilization of the unsaturated rings. By contrast, the aromatic stabilization of group 13 unsaturated rings results in a somewhat lower RSE than their saturated counterparts. In the case of group 15 and 16 heterocycles, the RSE of the unsaturated rings is much higher than that of their saturated counterparts due to the additional antiaromaticity destabilization effect.

### 2*H*-Unsaturated Three-Membered Rings **2**

**Scheme 2:**
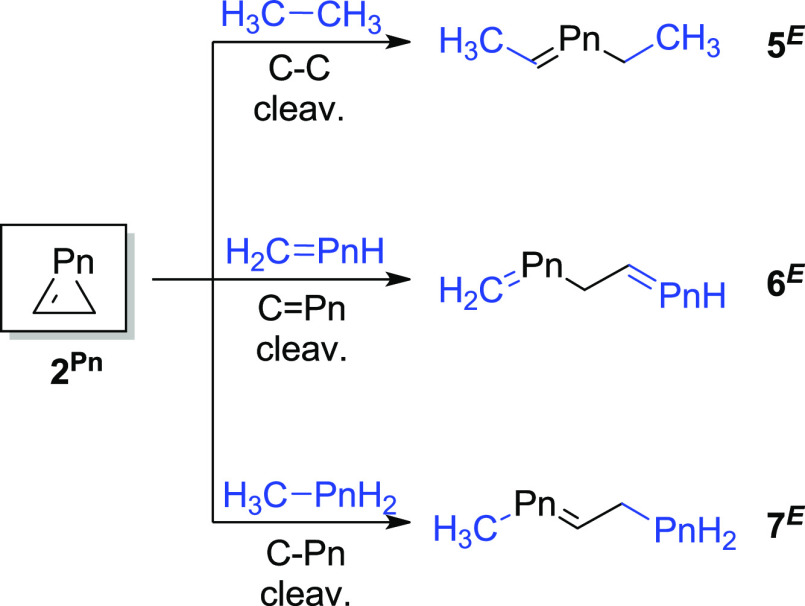
Homodesmotic (RC4) Ring-Opening Reactions Used for the Estimation of RSE for **2**^**Pn**^

2*H*-Unsaturated three-membered heterocycles (**2**^**El**^) were only found as energy minima for the elements of group 15 (**2**^**Pn**^). The RSE data for these pnictogen-containing rings (Table 2) were calculated using the same type of homodesmotic reaction scheme as for the previously described rings (Scheme 2).

**Figure 9:**
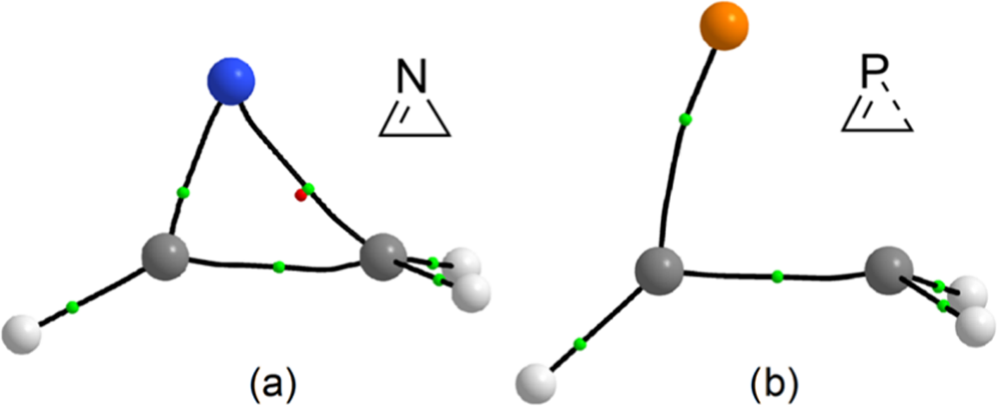
Computed (B3LYP/def2-TZVPP) BCP (small orange spheres) and bond paths for (a) **2**^**N**^ and (b) **2**^P*^.

However, only the 2*H*-azirine ring, **2**^**N**^, contains three BCPs corresponding to the three endocyclic bonds and a proper RCP (Figure 9a), while neither BCPs for the single C–El bond nor RCP were found for the rest of rings with heavier pnictogens. Taking **2**^P*^ as a case in point, the lack of RCP and BCP(P–C) can be observed (Figure 9b); furthermore, **2**^P*^ features a very elongated single C–P bond (1.906 Å) in comparison to the C–P single bond of the 1*H*-phosphirene **1**^**P**^ isomer (1.840 Å) or to that in phosphirane (1.876 Å).^25^ Consequently, except 2*H*-azirine (**2**^**N**^), all heavier 2*H*-pnictogenirenes **2**^Pn*^ must be considered pseudocyclic structures and their computed RSE used with caution. The analysis of the multireference RSE data calculated for these pseudorings (Table 2) shows that there is no significant difference with the single-reference (sr) data, even for **2**^Bi*^ (84.3% sr) and **2**^Sb*^ (86.5% sr) with a multireference contribution of about 15%.^54^

Recently, we have reported on the C=N bond-containing three-membered ring oxazirine.^34a^ The latter could be considered either as oxa-analogue (replacement of −CH_2_– by −O−) of 2*H*-azirine (**2**^**N**^), compared to which exhibits a somewhat enhanced RSE (44.28 kcal/mol), or as aza-analogue (replacement of =CH– by =N−) of pseudo-1*H*-oxirene (**1**^O*^), displaying similar pseudocyclic structure due to the absence of RCP as a consequence of the elongated N–O bond (with no BCP).

It is worth mentioning the higher stability of the 2*H*-pnictogenirene rings **2**^**Pn**^ compared to the corresponding 1*H*-isomers **1**^**Pn**^, as can be inferred from the variation in RSE (Table 2). This difference is very pronounced for the N-containing rings (ΔΔ*E*_ZPE_ = 29.83 kcal/mol), mainly due to the antiaromatic character of **1**^**N**^. For heavier pnictogens, this difference becomes less noticeable (ΔΔ*E*_ZPE_ = 5.46, 3.16, 0.04, and −1.20 kcal/mol for P, As, Sb, and Bi, respectively), probably due to the decrease of the antiaromatic character.

## Experimental Section

Density functional theory (DFT) calculations were performed with the ORCA program.^55^ All geometry optimizations were run in redundant internal coordinates in the gas phase, with tight convergence criteria, and using the B3LYP^56^ functional together with Ahlrichs segmented def2-TZVP basis set^57^ and the 2010 Grimme’s semiempirical atom-pairwise London dispersion correction (DFT-D3).^58^ From these geometries, all electronic data were obtained through single-point calculations (SP) using the same quality basis set but including additional polarization, def2-TZVPP.^59^ Energy values were corrected for the zero-point vibrational term at the optimization level and obtained by the newly developed DLPNO method^60^ for the “coupled-cluster” level with single, double, and triple perturbatively introduced excitations (CCSD(T)).^61^ It is worth mentioning that the RI-JK speeding up approximation for Coulomb and Hartree–Fock (HF) exchange (in HF step for post-HF calculations) gave rise to anomalous results for fifth-row elements even when using appropriate auxiliary basis sets.^25^ Therefore, this approach was skipped for all high-level RSE calculations. T1-Diagnostic values close to 0.02 prompted us to use dynamic correlation on top of the CASSCF(*n*,*m*) method^62^ employing a (6,6) active space for **1**^In*^ and **1**^O*^, whereas (8,6) for **2**^As*^, **2**^Sb*^ and **2**^Bi*^, with the multireference average coupled-pair functional (MRACPF)^63^ approach and the def2-SVPD basis set.^64^ Analysis of the hybridization in the AO used for the endocyclic bonds was performed with the NBO method.^65^ Properties derived from the topological analysis of the electronic density were obtained with the Multiwfn program,^66^ whereas Figures 2b, 4, 7, and 9 were plotted with AIMall.^67^Figure 3 was drawn with Visual Molecular Dynamics (VMD).^68^

## Conclusions

Accurate high-level (DLPNO-CCSD(T)/def2TZVPP//B3LYP-D3/def2TZVP) values were provided for the ring strain energy (RSE) in unsaturated three-membered rings containing only one 13–16 group element. A thorough exploration of the structural and electronic features of the 1*H*-unsaturated rings **1**^**El**^ resulted in the classification of those with the most metallic elements, **1**^Tl*^ and **1**^Pb*^, as pseudocyclic structures because of their typical Dewar–Chatt–Duncanson-type bonding lacking a ring critical point (RCP), 1*H*-indirene (**1**^In*^) constituting a borderline case. At the working level of theory, the controversial *C*_2*v*_-symmetric 1*H*-oxirene, **1**^**O**^, is a transition state (TS) between two low-symmetry (*C_s_*) degenerate minima (**1**^O*^) with pseudocyclic character. Among the 2*H*-unsaturated three-membered rings, **2**^**El**^, only found as a minimum for the pnictogen elements and presenting low RSE compared to the **1**^**El**^ isomers, just 2*H*-azirine (**2**^**N**^) presents a proper ring structure (*i.e.*, featuring an RCP).

RSE in groups 15 and 16 containing 1*H*-unsaturated rings **1**^**El**^ is very much affected by the strain relaxation effect of the increased s character of one LP orbital of pnictogens and chalcogens, as already described for their saturated counterparts. The existence of an empty orbital in group 13 rings gives them the possibility of presenting a significant aromatic character, especially in the case of borirene **1**^**B**^. The existence of empty d orbitals in 1*H*-tetrelirenes, allows their use to acquire some aromaticity, especially in the case of 1*H*-silirene **1**^**Si**^, while moving down in the group the energy of these d orbitals increases, hence decreasing this aromatic contribution. On the other hand, the high antiaromaticity of **1**^O*^ and **1**^**N**^, partially alleviated by geometric distortion, results in enhanced RSE. For the heavier group 15 and 16 elements, the antiaromaticity considerably decreases on descending the group, which together with the increase in heteroatom size and aromatic and/or hyperconjugative contributions result in a remarkable RSE decrease.

## Supporting Information Available

Plots of the relaxed force constants and of the RSE *versus* other parameters; additional tables with electronic, structural, and bond strength parameters; as well as Cartesian coordinates and energies for all calculated species (PDF)
