# Supplementary material for: Accurate Ring Strain Energies of Unsaturated Three-Membered Heterocycles with One Group 13–16 Element
Source: Inorg Chem. 2022 Apr 20;61(17):6459–68. doi: 10.1021/acs.inorgchem.2c00067 (PMC9066411; doi:10.1021/acs.inorgchem.2c00067)
Supplement: Supplementary file 1 — ic2c00067_si_001.pdf [file ic2c00067_si_001.pdf]

Supporting Information

Accurate Ring Strain Energies of Unsaturated Three-Membered Heterocycles with One Group 13−16 Element

Alicia Rey Planells[a] and Arturo Espinosa Ferao*[a]

[a] Depto. Química Orgánica, Facultad de Química, Campus de Espinardo, Universidad de Murcia, 30100 Murcia (Spain), E-mail: artuesp@um.es.

Table of contents

Figure S1. Computed (B3LYP/def2-TZVPP) structure with BCP and bond paths for $\mathbf{1}^{\mathbf{Tl*}}$. S2

Figure S2. Plot of $k^0_{C\text{-}El}$ *vs* p-character (%) used by "El" in C-El bond in **1**. S2

Figure S3. Plot of angular deviation for the C-El bond *vs* p-character (%) used by "El" in C-El bond in **1**. S2

Figure S4. Plot of RSE vs p-character (%) used by "El" for C-El bonds in **1**. S3

Figure S5. Computed (B3LYP/def2-TZVP) Kohn–Sham isosurfaces for $\mathbf{1}^{\mathbf{Si}}$. S3

Figure S6. Plot of RSE vs computed (B3LYP/6311+G(d,p) or def2-TZVPP) NICS(2) for **1.** S3

Figure S7. Plot of RSE vs NLMO-$BO_{C=C}$ for compounds **1**. S4

Table S1. Calculated magnetic, bond length and bond strength parameters for $\mathbf{1}^{\mathbf{El}}$ and $\mathbf{2}^{\mathbf{El}}$. S5

Table S2. Calculated (B3LYP-D3/def2-TZVPecp) C-El bond distances (Å) for $\mathbf{3}^{\mathbf{El}}$ and $\mathbf{4}^{\mathbf{El}}$. S6

Table S3. T1 diagnostic, single-reference contribution (%) and RSE values at the CCSD(T)(fc)/def2-TZVPP and CASSCF(n,m)/MRACPF/def2-SVPD levels. S6

Calculated structures. Cartesian coordinates and energies for all computed TS and minima. S7-S40

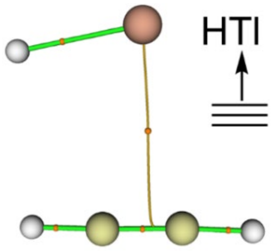

Figure S1. Computed (B3LYP/def2-TZVPP//B3LYP/def2-TZVP) structure with BCP (small orange spheres) and bond paths for pseudo-thallirene (**1**^{Tl*}).

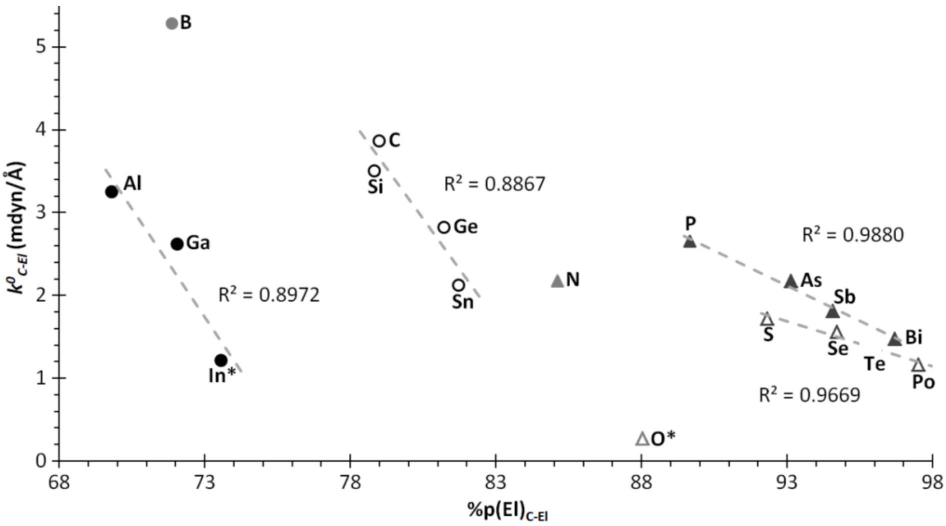

Figure S2**.** Plot of $k^0_{C\text{-}El}$ vs p-character (%) of AO used by heteroatom "El" in C-El bond in **1**.

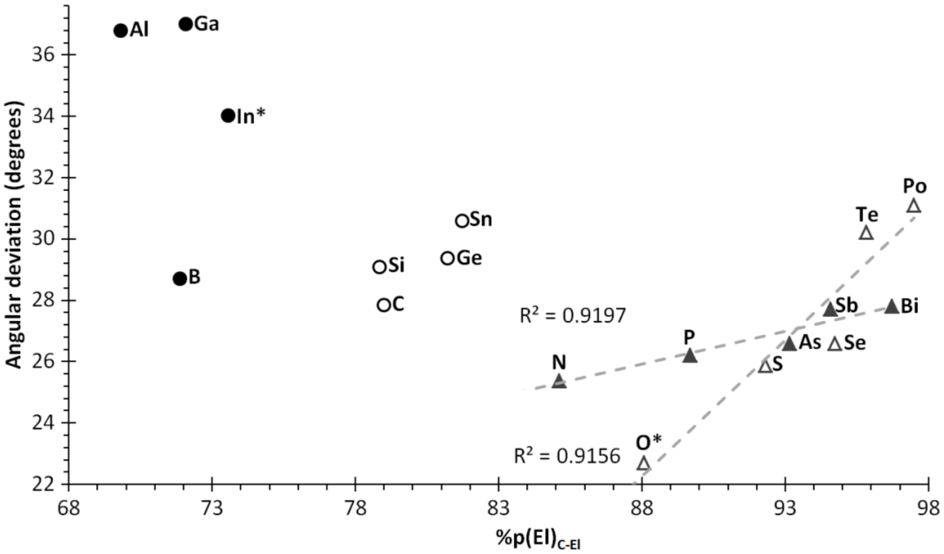

Figure S3. Plot of angular deviation of the C-El bond vs p-character (%) of the AO used by heteroatom "El" in C-El bond in **1**.

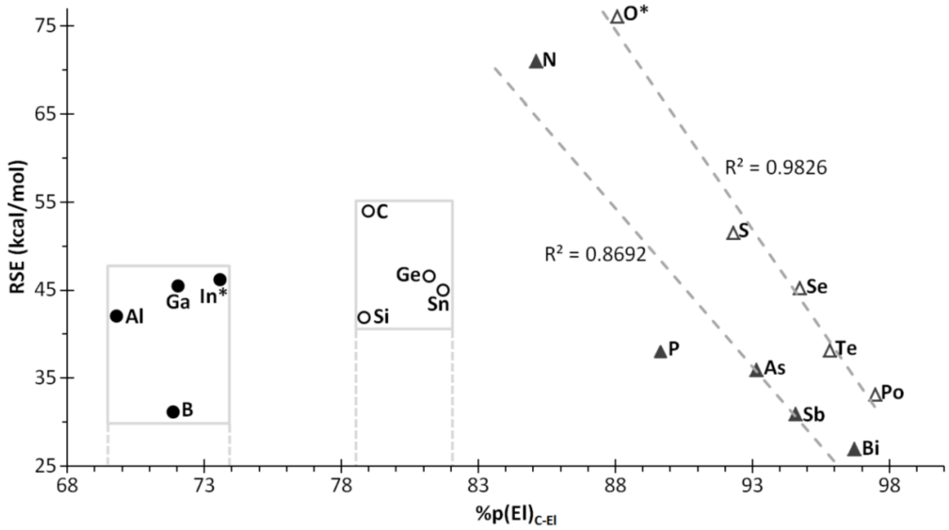

Figure S4. Plot of RSE vs p-character (%) of AO used by the heteroatom "El" for its endocyclic C-El bonds in **1**.

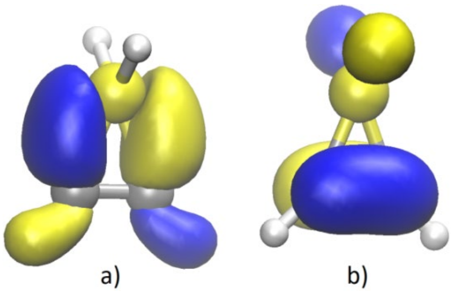

Figure S5. Computed (B3LYP/def2-TZVP) Kohn–Sham isosurfaces (0.07 au) for a) HOMO and b) HOMO-1 of $\mathbf{1}^{\mathbf{Si}}$**.**

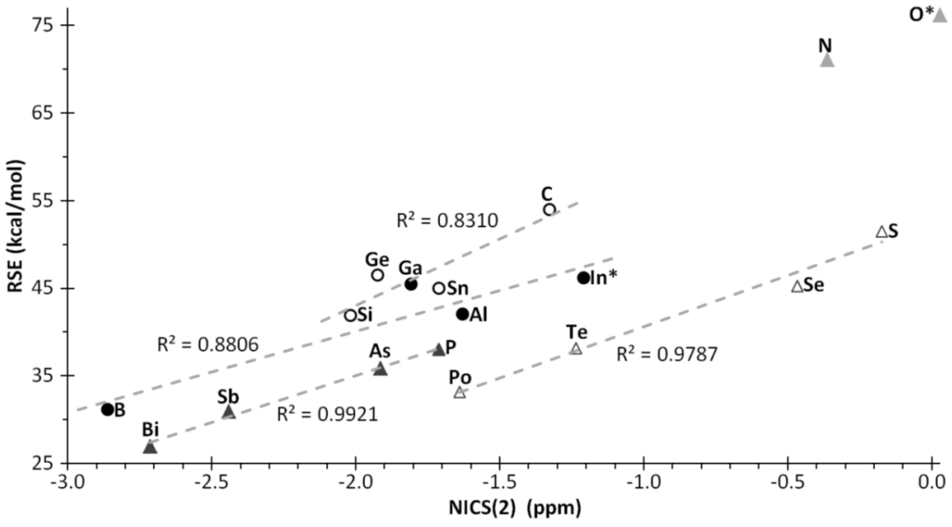

Figure S6. Plot of RSE vs computed (B3LYP/6311+G(d,p) or def2-TZVPP) NICS(2) (ppm) for compounds **1**.

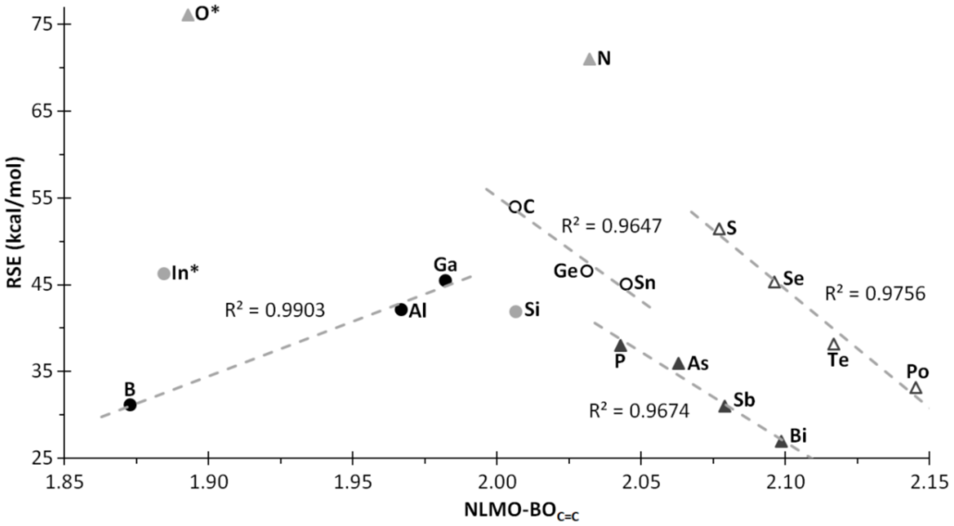

Figure S7. Plot of RSE vs NLMO-BO$_{C=C}$ for compounds **1** (**1^{In*}**, **1^{Si}**, **1^{N}** and **1^{O*}** are excluded from the linear correlation).

<u>Table S1</u>. Calculated magnetic, bond length and bond strength parameters for compounds **1**^{El} and **2**^{El}.

	NICS(1) a)	NICS(1)$_{zz}$ a)	d$_{C-C}$ c)	NLMO-BO$_{CC}$	WBI$_{CC}$	(G/ρ)$_{CC}$ b)	d$_{C-El}$ c)	NLMO-BO$_{C-El}$	WBI$_{C-El}$	(G/ρ)$_{C-El}$ b)	(G/ρ)$_{RCP}$ b)
**1**^{B}	-14.38	-26.33	1.346	1.873	1.720	0.500	1.468	1.035	1.146	1.301	1.454
**1**^{Al}	-9.35	-10.82	1.369	1.967	1.945	0.428	1.855	0.598	0.779	1.425	1.653
**1**^{Ga}	-8.80	-11.18	1.356	1.982	1.951	0.423	1.899	0.677	0.843	1.096	1.256
**1**^{In*}	-6.26	-22.25	1.330	1.885	2.028	0.442	2.210, 2.063	0.507, 0.818	0.680, 0.846	0.815	-
**1**^{C}	-6.94	-16.19	1.287	2.006	1.968	0.557	1.505	1.021	0.981	0.458	0.852
**1**^{Si}	-9.82	-16.09	1.33	2.007	1.982	0.477	1.812	0.696	0.883	1.261	1.463
**1**^{Ge}	-8.53	-14.36	1.319	2.031	2.035	0.472	1.923	0.721	0.871	0.907	1.101
**1**^{Sn}	-7.45	-9.90	1.320	2.045	2.067	0.458	2.118	0.704	0.845	0.876	1.095
**1**^{N}	-2.18	-9.38	1.269	2.032	2.021	0.566	1.517	0.858	0.941	0.601	0.954
**1**^{P}	-7.42	-12.80	1.292	2.043	2.046	0.520	1.840	0.852	0.927	0.719	1.007
**1**^{As}	-8.49	-14.85	1.287	2.063	2.097	0.515	1.985	0.849	0.889	0.647	0.882
**1**^{Sb}	-9.81	-16.52	1.287	2.079	2.137	0.507	2.180	0.816	0.850	0.744	0.957
**1**^{Bi}	-10.83	-18.25	1.280	2.099	2.179	0.513	2.293	0.841	0.820	0.738	0.938
**1**^{O*}	-0.66	-4.33	1.269	1.893	1.961	0.545	1.673, 1.376	0.596, 0.809	0.875, 1.014	0.108, 0.795	-
**1**^{S}	-2.01	-4.01	1.271	2.077	2.105	0.544	1.855	1.000	0.922	0.483	0.753
**1**^{Se}	-4.18	-8.23	1.268	2.096	2.148	0.541	2.008	1.011	0.882	0.559	0.784
**1**^{Te}	-6.96	-11.92	1.267	2.117	2.196	0.537	2.206	0.963	0.837	0.644	0.846
**1**^{Po}	-9.29	-15.69	1.261	2.145	2.247	0.549	2.324	0.984	0.786	0.701	0.858
**2**^{N}	-	-	1.447	1.034	1.020	0.443	1.246, 1.547	1.751, 0.914	1.968, 0.941	1.209, 0.730	0.804
**2**^{P}	-	-	1.477	1.064	1.104	-	1.637, 1.906	1.667, 0.816	1.852, 0.915	-	-
**2**^{As}	-	-	1.464	1.073	1.139	-	1.765, 2.058	1.656, 0.823	1.822, 0.901	-	-
**2**^{Sb}	-	-	1.456	1.079	1.181	-	1.972, 2.272	1.603, 0.820	1.766, 0.883	-	-
**2**^{Bi}	-	-	1.445	1.087	1.208	-	2.067, 2.385	1.623, 0.842	1.752, 0.872	-	-

a) In ppm; b) in a.u.; c) in Å.

Table S2. Calculated (B3LYP-D3/def2-TZVPecp) C-El bond distances (Å) for homodesmotic ring opening compounds $\mathbf{3}^{El}$ and $\mathbf{4}^{El}$

El	B	Al	Ga	In	C	Si	Ge	Sn	N	P	As	Sb	Bi	O	S	Se	Te	Po
$\mathbf{3}^{El}$	1.541	1.942	1.961	2.153	1.503	1.865	1.952	2.143	1.378	1.831	1.96	2.155	2.254	1.364	1.755	1.906	2.108	2.218
$\mathbf{4}^{El}$	1.557	1.956	1.978	2.173	1.498	1.869	1.956	2.153	1.387	1.817	1.964	2.164	2.267	1.368	1.752	1.907	2.112	2.224

Table S3. T1 diagnostic, single reference (SR) contribution (%) of and RSE values (kcal/mol) at the CCSD(T)(fc)/def2-TZVPP and CASSCF(n,m)/MRACPF/def2-SVPD levels (in parentheses).

	T1 diagnostic	% SR	RSE	(n,m)
$\mathbf{1}^{In*}$	0.02	76.2	46.22 (46.29)	(6,6)
$\mathbf{1}^{O*}$	0.02	80.6	76.10 (71.31)	(6,6)
$\mathbf{2}^{P*}$	0.01	92.1	32.57 (31.38)	(8,6)
$\mathbf{2}^{As*}$	0.01	89.9	32.73 (32.95)	(8,6)
$\mathbf{2}^{Sb*}$	0.02	86.5	30.91 (29.71)	(8,6)
$\mathbf{2}^{Bi*}$	0.02	84.3	28.12 (28.11)	(8,6)

Calculated structures

Cartesian coordinates (in Å), G correction (G-E) and ZPE (in hartrees) for TSs and minima were computed at B3LYP-D3/def2-TZVP. For TSs the imaginary frequency is given. In addition, electronic energies (in hartrees) are quoted using, unless otherwise indicated, the default DLPNO-CCSD(T)/def2-TZVPP(ecp) level.

1^{B} E = -102.582081476221 au
ZPE = 0.04177933 au
G_{corr} = 0.01807330 au

C	-0.11191044643341	0.46639729322614	-0.00073380806013
C	1.23427565549959	0.46707202659094	0.02160881680278
H	-0.92566216858447	-0.24384292185887	-0.00039014259886
H	2.04821220830688	-0.24242336551970	0.04906718609662
B	0.56094812021935	1.77074871915449	-0.01454097430694
H	0.56077414099206	2.94839776840700	-0.03736605793348

1^{Al} E = -319.776580353796 au
ZPE = 0.03606319 au
G_{corr} = 0.01090857 au

C	-0.12309096995491	0.31259316484217	0.00290506679181
C	1.24544895784992	0.31323794088778	0.02561579891488
H	-0.78663156170170	-0.54899421723522	0.00725376853558
H	1.90931775528607	-0.54769865797809	0.05209414244392
Al	0.56093008727606	2.03696242113579	-0.02044381583626
H	0.56066324124457	3.60024886834756	-0.04977994084993

1^{Ga} E = -2001.232306587126 au
ZPE = 0.03535385 au
G_{corr} = 0.00893323 au

C	-0.11662457061814	0.28910816143441	0.00331589615433
C	1.23900452054553	0.28971519022661	0.02583610357724
H	-0.82867951686637	-0.53372785597887	0.00632589026088
H	1.95135667296059	-0.53243144275871	0.05255327073051
Ga	0.56095372132760	2.06290418490378	-0.02042472310225
H	0.56062668265078	3.59078128217278	-0.04996141762072

1^{In*} E = -267.231457619510 au
E = -266.913253182 au [CASSCF(6,6)/MRACPF/def2-SVPD]
ZPE = 0.03345178 au

G_{corr} = 0.00560013 au

C	-0.14962067686739	0.22891367097567	0.00324025923304
C	1.17963899832784	0.17792521014688	0.02614643787168
H	-0.82425496573299	-0.62935232648408	0.00894802786358
H	1.96314055416247	-0.57391946339259	0.05407678806407
In	0.82848242912478	2.21054595205977	-0.01868478554529
H	0.17437266098530	3.75211795669432	-0.05932372748706

1^{Tl*} E = -249.632634473190 au
ZPE = 0.03165075 au
G_{corr} = 0.00131178 au

C	-0.01051095576705	0.02096002764612	0.07977972989978
C	1.14750457680096	-0.29917120520691	0.08311440431922
H	-1.02488617664198	0.34070307047558	0.07437130370805
H	2.15731811204406	-0.63227564176242	0.08905043188202
Tl	1.41315132339879	2.79997596071200	-0.17000211993674
H	-0.51081787983476	2.93603878813557	-0.14191074987231

1^{C} E = -116.398803235302 au
ZPE = 0.05581697 au
G_{corr} = 0.03187517 au

C	1.86704377958438	-1.50077910912250	1.45358489549942
C	3.36721635819531	-1.37790302148779	1.45302743170999
C	2.72419604939889	-0.26295099792165	1.45224002670173
H	1.35040170210964	-1.79934739339877	0.54238974427655
H	1.35092628697348	-1.79771344465639	2.36561096767832
H	2.72945614885779	0.81222240989786	1.45127143933111
H	4.30093764488050	-1.91087148331075	1.45323349480288

1^{Si} E = -367.441840561261 au
ZPE = 0.04658363 au
G_{corr} = 0.02136082 au

C	3.48121315585138	-1.33741043137068	1.45364718082077
C	2.81601593686222	-0.18517154337596	1.45145848441966
H	3.08300251991876	0.86322985313610	1.44967458856938
H	4.52274032756038	-1.62991416532628	1.45437181858347
Si	1.68917917194463	-1.60393497825800	1.45372383540273
H	0.96516775407433	-2.01753894086578	2.67414407137363
H	0.96620769378830	-2.02277540393939	0.23447245083035

1^{Ge} E = -2153.978124181148 au
ZPE = 0.04436970 au
G_{corr} = 0.01781850 au

C	3.52469219041442	-1.30592189334541	1.45368779711994
C	2.86519195766482	-0.16331888288164	1.45133406578101
H	3.10186522378521	0.89302322304515	1.44930226768681
H	4.55800911470969	-1.62844549477169	1.45470614540399
Ge	1.63015809658425	-1.63748209356681	1.45377081174596
H	0.92132277957689	-2.04302912487032	2.74896048318416
H	0.92228719726469	-2.04834134360927	0.15973085907812

1^{Sn} E = -292.010339573505 au
ZPE = 0.04130559 au
G_{corr} = 0.01352533 au

C	3.60883922299819	-1.25772530503299	1.45382182132178
C	2.94919572496598	-0.11488169222410	1.45105841940529
H	3.22317219842184	0.93458986414983	1.44875452494755
H	4.65498453489044	-1.54393052374490	1.45506786311442
Sn	1.53625444542211	-1.69164415794299	1.45385808579463
H	0.77484620005513	-2.12674275325932	2.90890298706189
H	0.77623423324630	-2.13318104194554	0.00002872835444

1^{Pb*} E = -270.558650246835 au
ZPE = 0.03823709 au
G_{corr} = 0.00757307 au

C	3.70642123668768	-1.04668114553044	1.43681560412726
C	3.35518865632941	0.10373212652914	1.46421663377336
H	3.12823166498744	1.14293556509305	1.48868187438088
H	4.01797441450439	-2.06386086031412	1.41293403005530
Pb	0.76464365956564	-1.22337874433388	1.42760793555643
H	1.24362375382011	-2.38942550307899	2.79161900886110
H	1.30744317410526	-2.45683704836473	0.14961734324561

1^{N} E = -132.397516252899 au
ZPE = 0.04338520 au
G_{corr} = 0.01942865 au

C	-0.17792634338506	0.24249136300042	-0.29389510082375
C	1.06113242286821	-0.02354782467462	-0.36393680672687
H	-1.22800301389374	0.07112304597715	-0.42857771710970
H	1.92675146723146	-0.60747875279581	-0.60856095613264
N	0.71698190139017	1.16298153723927	0.51509797018401
H	0.86812469578895	2.01185553125358	-0.03875801939106

1[P] E = -418.685417516564 au
ZPE = 0.03844776 au
G_{corr} = 0.01331884 au

C	-0.20496171222566	0.16088010016102	-0.25402686415296
C	1.05633028765661	-0.11056130943616	-0.32593836145074
H	-1.18683195864658	-0.12928550480846	-0.58930158076198
H	1.79007045515649	-0.76975277718835	-0.75918856332947
P	0.76443634003677	1.30573407806731	0.81184078302035
H	0.94801771802236	2.40041031320462	-0.10201604332519

1[As] E = -2312.245219181592 au
ZPE = 0.03673259 au
G_{corr} = 0.01032560 au

C	-0.21295714576434	0.11490499977754	-0.26694857438124
C	1.04328330570080	-0.15535674444312	-0.33830578722472
H	-1.19296581795149	-0.16361500615727	-0.61854133338603
H	1.77840233511935	-0.80240342214903	-0.78832051876174
As	0.78218590750682	1.36329559332317	0.91201118448736
H	0.96911254538884	2.50059947964869	-0.11852560073363

1[Sb] E = -317.377830937677 au
ZPE = 0.03503248 au
G_{corr} = 0.00770270 au

C	-0.22653141315982	0.05554275210001	-0.28208719842160
C	1.02984311717281	-0.21497141995898	-0.35335111113291
H	-1.19044282091545	-0.24082611982189	-0.66670150702413
H	1.73898921125031	-0.87143033061100	-0.83379158451022
Sb	0.80697668135818	1.43829664386362	1.04923463893375
H	1.00822635429395	2.69081337442822	-0.13193386784488

1[Bi] E = -291.736818748145 au
ZPE = 0.03401879 au
G_{corr} = 0.00576423 au

C	-0.23260327247534	0.01435850652234	-0.29753821013711
C	1.01699090985678	-0.25430348856188	-0.36851988148050
H	-1.20287781698860	-0.26012271067157	-0.68112627728443
H	1.74074802958943	-0.89355253522220	-0.84939977111236
Bi	0.81930123303258	1.47956380675544	1.11876487046327
H	1.02550204698514	2.77148132117785	-0.14081136044887

1^{O*} E = -152.234397343508 au
E = -151.978189782078 au [CASSCF(6,6)/MRACPF/def2-SVPD]
ZPE = 0.02856521 au
G_{corr} = 0.00338652 au

C	-0.16876074341669	0.36831172164484	0.00322103919356
C	1.08186885785998	0.58525644893826	0.00322265224104
O	0.57829609148528	1.86529647475980	0.00322331488164
H	-1.20609363281931	0.12968610730920	0.00322330638143
H	2.09496742689076	0.21748224734791	0.00322368730232

1^{O}(C_{2v}) E = -152.235384171971 au
ZPE = 0.02879446 au
G_{corr} = 0.00338652 au
v=-133.17 cm^{-1}

C	-0.12595178787477	0.49679426908575	0.00322297882492
C	1.13387951334934	0.49292838093524	0.00322301571306
O	0.50800051021334	1.85398958075426	0.00322274062212
H	-1.14342506690123	0.16598584060224	0.00322268995408
H	2.14931015121333	0.15589187862251	0.00322267488583

1^{S} E = -474.893167668796 au
ZPE = 0.02891103 au
G_{corr} = 0.00380176 au

C	-0.13147393853800	0.48875825381917	0.00322292546310
C	1.13931682797716	0.48491295709270	0.00322284757778
S	0.50922021157633	2.22962117903168	0.00322279643068
H	-1.07965983034874	-0.01402742696141	0.00322274934408
H	2.08441004933326	-0.02367501298214	0.00322278118436

1^{Se} E = -2477.288891647312 au
ZPE = 0.02832749 au
G_{corr} = 0.00192120 au

C	-0.13033311691047	0.46077157592779	0.00322184621589
C	1.13801024153964	0.45692731805563	0.00322179685741
Se	0.50961688099455	2.36394570181158	0.00322328431086
H	-1.07289874522778	-0.05321833338200	0.00322357623554
H	2.07741805960407	-0.06283631241300	0.00322359638029

1^{Te} E = -344.619241498200 au
ZPE = 0.02781396 au
G_{corr} = 0.00055766 au

C	-0.13003837805628	0.43088299070273	0.00322284405750
C	1.13745297323640	0.42703434364600	0.00322287436900
Te	0.51025302560422	2.54234181899867	0.00322285740351
H	-1.05720365665606	-0.11263908329409	0.00322276838343
H	2.06134935587173	-0.12203012005330	0.00322275578655

1^{Po} E = -314.339731777111 au
ZPE = 0.02745950 au
G_{corr} = -0.00062366 au

C	-0.12680158251231	0.39837061425890	0.00322317530870
C	1.13403430281097	0.39442833401429	0.00322339126551
Po	0.51046838528977	2.63336673202051	0.00322270127730
H	-1.06443857322698	-0.12554302041392	0.00322246093980
H	2.06855078763856	-0.13503270987979	0.00322237120869

2^{N} E = -132.452255412637 au
ZPE = 0.04479942 au
G_{corr} = 0.02100420 au

C	1.03481393975417	1.47287790840700	-0.63468299774773
C	2.13374629681094	0.99650741640888	0.17739135601863
H	0.24946157466928	1.30953622034560	-1.36044065411421
H	3.12250741144054	0.87008619011323	-0.24951286651923
H	1.94676293193844	0.47557246751895	1.10996477280139
N	1.50765434538661	2.40507659720635	0.04342237956115

2^{P*} E = -418.696508729263 au
E = -418.425217363244 au [CASSCF(8,6)/MRACPF/def2-SVPD]
ZPE = 0.04158183 au
G_{corr} = 0.01648698 au

C	1.01244807341974	1.49874910547585	-0.64708467609665
C	2.13114738197466	0.99148167089641	0.17369218711902
H	0.31207364324383	1.10365033545254	-1.36568643884491
H	3.08023394914555	0.76051263462227	-0.30338408584318
H	1.91763954570488	0.37142080108080	1.04074470637057
P	1.54140390651134	2.80384225247212	0.18786029729515

2^{As*} E = -2312.253356272316 au

E = -2311.81745422462 au [CASSCF(8,6)/MRACPF/def2-SVPD]
ZPE = 0.04050448 au
G_{corr} = 0.01415139 au

C	1.01976035054711	1.48034694670512	-0.64640136391504
C	2.12553966580225	0.95826803931169	0.15929851192643
H	0.31998225503893	1.08044269748108	-1.36500134585186
H	3.07834576886473	0.73598351405513	-0.31328872888010
H	1.91416197351969	0.34560982499642	1.03154066976756
As	1.53715648622729	2.92900577745056	0.21999424695302

2^{Sb*} E = -317.379353178921 au
E = -317.035305908757 au [CASSCF(8,6)/MRACPF/def2-SVPD]
ZPE = 0.03912539 au
G_{corr} = 0.01179627 au

C	1.02109447523054	1.45963119557854	-0.64993853532623
C	2.11854450124462	0.92364453930302	0.14345459561598
H	0.33611340630008	1.02931293024868	-1.36731635256689
H	3.06882079349821	0.69464520065206	-0.33230152254381
H	1.90651445600869	0.30499941912605	1.01187883566456
Sb	1.54385886771785	3.11742351509163	0.28036496915639

2^{Bi*} E = -291.740202592561 au
E = -291.32937433135 au [CASSCF(8,6)/MRACPF/def2-SVPD]
ZPE = 0.03807973 au
G_{corr} = 0.00943765 au

C	1.02583007201814	1.44180694499991	-0.64811658863832
C	2.11381249109925	0.89731408214512	0.13214096405543
H	0.34051279524317	1.01824265427787	-1.37012168650612
H	3.06898673458904	0.67784770044399	-0.33842579103061
H	1.90753694297870	0.28932173870001	1.00937329792957
Bi	1.53826746407169	3.20512367943309	0.30129179419007

3^{B} E = -181.078398231603 au
ZPE = 0.09763374 au
G_{corr} = 0.06858279 au

C	2.40419483452755	2.42327227593686	-0.36123123989168
H	2.98987628979905	1.56434723793492	-0.67384253979683
H	2.96480982833797	3.29610797777745	-0.04287938580743
C	1.06447381162435	2.43038608398239	-0.35925815746323
H	0.55816679639598	1.52418003973644	-0.68965781635885
C	-1.28493338092460	3.70126621878966	0.10048727376542

C	-1.96550948244417	4.78561872430058	0.49587566759631
H	-1.86386239530407	2.83470017427933	-0.21678864255164
H	-1.44494818800271	5.67983044228891	0.82268836981057
H	-3.05014692795076	4.82866991312919	0.51103809751240
B	0.25572497055594	3.66371454452259	0.08800080319374
H	0.85321801338547	4.63958964732166	0.44305724999122

3^Al^ E = -398.287067103634 au
ZPE = 0.09024841 au
G_{corr} = 0.05939003 au

C	2.66907811661605	2.21987874900644	-0.43822773586084
H	3.27704552680778	1.37428523518002	-0.75172879448442
H	3.22676119866236	3.09683077819248	-0.11913830407778
C	1.33091140268192	2.19858893067814	-0.43959530976656
H	0.86235555826334	1.27430809968726	-0.77705488681140
C	-1.63067114818137	3.80129540147389	0.13933829970880
C	-2.28716018454514	4.89908192606241	0.53371939345717
H	-2.24480172234018	2.95570313916918	-0.16989468428834
H	-1.76227597003844	5.79358716751188	0.85945270635380
H	-3.37252888328273	4.96679095059358	0.55369268111337
Al	0.31093502770852	3.75123206723666	0.12715094546777
H	1.10141524764788	5.04010083520803	0.59977536918841

3^{Ga} E = -2079.747945523686 au
ZPE = 0.08994660 au
G_{corr} = 0.05835939 au

C	2.68369966251693	2.20711167437878	-0.45006695658854
H	3.27311462954541	1.35294948689372	-0.77462660463571
H	3.25527488180870	3.07704747039180	-0.13916909441725
C	1.34877789752304	2.19789211800370	-0.43162610820119
H	0.84797040871565	1.28900071677916	-0.75903863183872
C	-1.63760730646667	3.81591568829031	0.14837557096534
C	-2.30854191307850	4.90548474958607	0.53001397098240
H	-2.21995021625691	2.95290833590140	-0.16835865387535
H	-1.79922775450269	5.80697276633442	0.85856745225152
H	-3.39480451519115	4.95493236924607	0.53449362592463
Ga	0.32299029109535	3.76609405934286	0.14681658021226
H	1.10936810429084	5.04537384485167	0.62210852922060

3^{In} E = -345.747663869996 au
E = -345.292307955471 au [CASSCF(6,6)/MRACPF/def2-SVPD]
ZPE = 0.088177790au

G_{corr} = 0.05550770 au

C	2.81610097669133	2.10122346307007	-0.49070809509700
H	3.39272363755486	1.23895619783307	-0.81948701660638
H	3.40324934621498	2.96216782623892	-0.18422201439685
C	1.48279807238448	2.10251114070899	-0.46507133882907
H	0.97153949486624	1.19879730616910	-0.78943961352459
C	-1.79273781575351	3.87628513575037	0.17019877118814
C	-2.47259781655576	4.95983971555565	0.54779627354755
H	-2.36514877412539	3.00790275684488	-0.14857989364533
H	-1.97871333606964	5.86871793807448	0.87920987672045
H	-3.56017661384195	4.99617758976422	0.54591980483604
In	0.35924605111747	3.82453475977620	0.17228674739227
H	1.22478094751689	5.23456945021399	0.69958617841476

3^{C} E = -194.929925984928 au
ZPE = 0.11306921 au
G_{corr} = 0.08406477 au

C	-2.54641206957776	-0.07680558224637	-0.81946558120829
C	-2.21807386143828	-0.18579260854419	0.46063286690815
C	-0.82943550343075	-0.45287995021608	0.97113026039523
H	-0.14162434571924	-0.58651812228651	0.13318154198908
C	-0.33264348665355	0.64040374109966	1.87569974945566
C	0.75943898665382	1.36203819289727	1.66357289500391
H	-1.80567263927121	-0.18109877935432	-1.60475339120508
H	-3.56475330353127	0.11932846959989	-1.13017480513039
H	-2.98816222193389	-0.06872261311600	1.21936372498264
H	-0.93832848016502	0.83914162704316	2.75666310380281
H	1.38610217558222	1.19909665973863	0.79352905961580
H	1.06821353923068	2.14026846802876	2.34994612553646
H	-0.83940261974575	-1.39550005264391	1.53467301985403

3^{Si} E = -445.952046717619 au
ZPE = 0.10164456 au
G_{corr} = 0.07097764 au

C	-2.80055265846305	-0.07270300304693	-0.94137169920858
C	-2.45232948818175	-0.30623266093401	0.32277525862275
C	-0.12092470683034	0.71185301804415	2.07355618532567
C	0.89789840036287	1.53618275953111	1.83666786397926
H	-2.06499355789520	-0.03667639781637	-1.73873107306640
H	-3.83137398598643	0.09128209232233	-1.23918274710420
H	-3.24215694892508	-0.32960883711753	1.07172152713640
H	-0.64640950514159	0.81235523716500	3.02179643855168
H	1.46536464698700	1.49162743784754	0.91258787476689

H	1.21286028303342	2.29042295177551	2.55097414949899
Si	-0.69196410595006	-0.59235979378744	0.86892998489971
H	-0.59908346875509	-1.92531223507643	1.52084126907926
H	0.18973033574533	-0.56026985890694	-0.32421607248146

3[Ge] E = -2232.495275662887 au
ZPE = 0.09968239 au
G_{corr} = 0.06787856 au

C	-2.85521806978563	-0.06325486999967	-0.96031735793882
C	-2.50460648310169	-0.33206079334665	0.29295987175403
C	-0.07791333028049	0.73111051531865	2.11914097918619
C	0.91492873579271	1.57896249634310	1.87154283520153
H	-2.12546059862621	-0.00075031201795	-1.76124308572406
H	-3.88905338122055	0.10621933914363	-1.24530714354211
H	-3.27977988599268	-0.38321754724643	1.05351065978897
H	-0.60166229711060	0.80085235728196	3.06922502536736
H	1.47207843819758	1.55592850433187	0.94052124026120
H	1.21802517579829	2.33644260269209	2.58791353363167
Ge	-0.65844182493983	-0.63290605873909	0.85011066412814
H	-0.54809423187126	-2.02425662240337	1.51107115768191
H	0.25126299314039	-0.56250890135816	-0.39277941979602

3[Sn] E = -370.524753188627 au
ZPE = 0.09651669 au
G_{corr} = 0.06349551 au

C	-2.99736989164554	-0.07463054667329	-1.01872732139035
C	-2.64128752436871	-0.37211111186981	0.22657331560953
C	0.02885717031231	0.78003441094920	2.23574850617608
C	0.97930057144564	1.67226389177955	1.97775517638312
H	-2.27416832920996	0.02063595747949	-1.82271044987137
H	-4.03500452720735	0.08831042566366	-1.29751286832223
H	-3.41436896188181	-0.45457404017944	0.98595239437096
H	-0.45445623711672	0.79746910200260	3.20899054994829
H	1.50047383918685	1.70703318640575	1.02619344752932
H	1.28512779562621	2.41564176383440	2.70909111314621
Sn	-0.60301257604098	-0.68884335491556	0.80863225930426
H	-0.43501998925577	-2.25794360887392	1.48641957707503
H	0.37699390015588	-0.52272536560264	-0.59005673995889

3[N] E = -210.965067349400 au
ZPE = 0.10168069 au
G_{corr} = 0.07311990 au

C	0.98675746502561	-1.03454206374964	1.45979502706591
H	1.32812916353752	-2.05787306420403	1.46737002406874
H	-0.06250103828821	-0.85558452642333	1.25935788724015
C	1.84640913358232	-0.04003085583301	1.69287140753692
H	2.89211148083159	-0.24864288832011	1.88939694119932
N	1.54368194772229	1.30450068622711	1.71496863307261
H	0.58663893480434	1.57109863272912	1.54251243381101
C	2.45599597741152	2.31175177433899	1.94548487036831
C	2.19269277805249	3.62047070350761	1.96445264149996
H	2.98354000585743	4.32917413036673	2.15437985355574
H	1.19692533926385	4.01151969797515	1.79510638501193
H	3.46247607219922	1.94820685338539	2.11991085556939

3^P^

E = -497.189772615128 au
ZPE = 0.09433612 au
G_{corr} = 0.06405147 au

C	0.94786030883073	-1.08496396166422	1.97065359209497
H	1.31972419949421	-2.07209339265616	2.22207411684573
H	-0.09311695103563	-0.88277120547105	2.19809403364935
C	1.72532003822454	-0.16566783864749	1.40905455477943
H	2.76628104733093	-0.39826297146450	1.19839243535174
P	1.08788491411730	1.50083947784919	0.99768243736175
H	1.57971860376890	1.52765430808234	-0.33773393111847
C	2.41993977352055	2.52965303411432	1.71870482284035
C	2.14695429112598	3.58051537665357	2.48458504787388
H	2.93140510001940	4.19136196565572	2.91761542937033
H	1.12542307095654	3.87014044123464	2.70565166294247
H	3.45546286364651	2.26364384631361	1.52083275800843

3^{As}

E = -2390.745070386180 au
ZPE = 0.09225295 au
G_{corr} = 0.06072936 au

C	0.96660974159445	-1.17067137158354	1.98640907749322
H	1.37839199113560	-2.14532767727155	2.22687037893473
H	-0.07344417526060	-1.00785747521993	2.24857094509276
C	1.69583658941954	-0.22934937452821	1.40190915649858
H	2.73601435850394	-0.41849248137114	1.15354628490194
As	0.95056896908137	1.53639389817394	0.98944479508128
H	1.49520023840030	1.56170203909953	-0.44280506439246
C	2.42347506362306	2.59807879257297	1.73000871428412
C	2.19764771553099	3.64413625364785	2.51422160107839
H	3.00942536362753	4.22909704461330	2.93410304456958
H	1.19223106486005	3.96139331379059	2.77016906698954
H	3.44090033948373	2.30094611807618	1.49315895946827

3^{Sb} E = -395.869163598903 au
ZPE = 0.09007584 au
G_{corr} = 0.05743155 au

C	0.98054189750172	-1.29778496410922	2.01816309708753
H	1.43167417691859	-2.25824463926271	2.25040456269037
H	-0.05269022922400	-1.17290276278826	2.32650552651009
C	1.65436468536118	-0.33873622123833	1.39518985976787
H	2.68789337062212	-0.50598252020514	1.10571985134816
Sb	0.78010032488027	1.58187651324576	0.95720514861864
H	1.40186961174190	1.60161702687142	-0.64874316942979
C	2.43777603240288	2.71891873493552	1.73572171193709
C	2.26549704821305	3.73908461172654	2.56765382404646
H	3.10372002680524	4.30180555597328	2.96853532240968
H	1.28094087572130	4.06332432865649	2.89019892381246
H	3.44116943905570	2.42707341619463	1.43905230120138

3^{Bi} E = -370.220572137594 au
ZPE = 0.08876986 au
G_{corr} = 0.05510010 au

C	0.98490701522934	-1.35338538607844	2.02481915136942
H	1.45225633487051	-2.30879844002878	2.24973756650132
H	-0.04660599624411	-1.24864314728811	2.34777471927961
C	1.63712624508007	-0.38453248060590	1.39628639966312
H	2.66914784981422	-0.52915332873059	1.09108351705285
Bi	0.68596273741411	1.61089965366749	0.95526547860596
H	1.32972677914019	1.62146114816634	-0.73224047289609
C	2.45093942315219	2.78099049131769	1.72833303918177
C	2.30939978745906	3.76931440230033	2.60171191085800
H	3.16187669086245	4.32198824029707	2.98846620501324
H	1.33947543613509	4.08088982120689	2.97820180841373
H	3.43864495708685	2.49901810577600	1.37616763695704

3° E = -230.809390657099 au
E = -230.41145989472 au [CASSCF(6,6)/MRACPF/def2-SVPD]
ZPE = 0.08879633 au
G_{corr} = 0.06016918 au

C	0.94386128047325	-0.96958705976819	1.61160729402810
H	1.29167437805025	-1.98554085281176	1.50282894035687
H	-0.12081064558318	-0.78224472719105	1.62919191224570
C	1.82384773946367	0.01428843488731	1.72023107048584
H	2.89725075864048	-0.14864867080323	1.70636704894347

C	2.40769821759071	2.26958955704704	1.97135132744385
C	2.11491519657407	3.55536496407581	2.09483082933037
H	1.09171604656287	3.90433061510602	2.11164865134494
H	3.42639477009860	1.89442784739158	1.95392174682808
O	1.43870676946395	1.31586773012716	1.85771877846571
H	2.91241844866531	4.27732356193930	2.18233178052706

3^S^ E = -553.419919037401 au
ZPE = 0.08516251 au
G_{corr} = 0.05485164 au

C	0.99023330814283	-1.26839925212112	1.61677570912071
H	1.49882018274799	-2.21322309767550	1.48196137624903
H	-0.08437837133470	-1.29576508793037	1.74647144903797
C	1.67676501328958	-0.13034184726959	1.61032142164479
H	2.75302652950421	-0.12461466425381	1.47904711740198
C	2.33780570145435	2.47068492926449	2.05375482252005
C	2.29109857650027	3.79780200297334	2.10953407601341
H	1.37443122526448	4.35286376444053	1.95437680814953
H	3.26565149094007	1.93420426073709	2.21621615541242
S	0.93818225253131	1.45540721741541	1.75230964071798
H	3.18603705095958	4.36655317441952	2.32126080373212

3^Se^ E = -2555.804304186445 au
ZPE = 0.08386590 au
G_{corr} = 0.05169881 au

C	0.99950369207126	-1.36258713255009	1.58178778986285
H	1.55178522511005	-2.28381490858831	1.44672591833707
H	-0.07721894563793	-1.44295054684306	1.66666154287376
C	1.63647366376877	-0.20012950113502	1.63353957375664
H	2.71484592303972	-0.13767104278169	1.54531965212505
C	2.33577910790006	2.55024773246091	2.07832760788524
C	2.34156879272376	3.87645442187615	2.10177593141268
H	1.44611705441976	4.46774531335142	1.95558283315124
H	3.24150235310378	1.97424839013333	2.22796488169907
Se	0.77335517148645	1.48774456615018	1.82901460438601
H	3.26396092201430	4.41588410792618	2.27532904451037

3^Te^ E = -423.121865330448 au
ZPE = 0.08294766 au
G_{corr} = 0.05146784 au

C	0.99952530024462	-1.42830160450882	1.72722877264346
H	1.52759930717342	-2.37371361397898	1.78728657778055

H	-0.06877157249389	-1.45707961584501	1.90550604340456
C	1.64763201479456	-0.30553399085275	1.44756214748104
H	2.71925939965413	-0.30103195897515	1.27898070025959
C	2.36701472517741	2.65752434649623	1.92162222684534
C	2.34396374403055	3.93677715507248	2.27058170606328
H	1.44476022515038	4.54022472115316	2.23090271209450
H	3.28021486257978	2.07501568199071	1.98052474447421
Te	0.72241623730276	1.56885910802409	1.17557691076972
H	3.24405871638625	4.43243117142402	2.61625683818371

3^Po^ E = -392.833776529030 au
ZPE = 0.08255021 au
G_{corr} = 0.04932501 au

C	1.08060296348562	-1.50537990402885	1.60288846619737
H	1.63141931892059	-2.39313695591450	1.30695738781876
H	0.13602994344768	-1.67103390871020	2.10833242970933
C	1.55418075408888	-0.29375101459021	1.34991024572304
H	2.50424389704462	-0.14315297747621	0.85059559803029
C	2.26726968107076	2.68785164354636	2.38498119703191
C	2.48672852327018	3.95465858116237	2.06462100094388
H	1.82039181263823	4.51880648332441	1.42220291409935
H	2.94492051841800	2.13230615245982	3.02290124464103
Po	0.44458767870543	1.57492048285570	1.78730187216331
H	3.35729786890998	4.48308281737129	2.44133702364168

4^B^ E = -168.432616101051 au
ZPE = 0.10168855 au
G_{corr} = 0.07107503 au

C	2.45507116855023	2.39445242570652	-0.45860055030942
H	2.95688810512955	3.35212448174567	-0.32561081019476
C	1.11704070397972	2.37500132124497	-0.25458611668786
H	0.61506173320720	1.41560047621762	-0.38730916561071
B	0.28480550084264	3.64654488255976	0.08558169480331
H	0.89342718044325	4.63795030982370	0.36938228558193
B	3.24938606715277	1.14558693962105	-0.90379297013982
H	4.40496072759847	1.20998876131264	-1.19122414489473
H	2.68769676616133	0.09432817120664	-0.96595389397156
C	-1.27221435965075	3.66592196054733	0.10304163460644
H	-1.75271639460624	2.80803391538831	-0.37116359403590
H	-1.69158535860168	4.58978183883253	-0.30423238624709
H	-1.57491163020649	3.66249278579320	1.16225197710018

4^{Al} E = -602.863432105516 au
ZPE = 0.08681485 au
G_{corr} = 0.05296906 au

C	2.58788960101943	2.39757317656764	-0.56185253815956
H	3.03514070756042	3.39073605509347	-0.47206689884688
C	1.25058865586774	2.31015586377423	-0.37598673725026
H	0.80517617428282	1.31566896018487	-0.46638456740210
Al	0.17353981080503	3.89079160855878	0.03485898755383
H	0.97738353203699	5.25397386847370	0.14420358797229
Al	3.65956271485132	0.82257373850741	-0.97684655702306
H	5.22304328033866	0.90610344924355	-1.21676934663511
H	2.89322601881220	-0.55990022461901	-1.06707264011190
C	-1.76058423463924	3.82101242439340	0.32198489968099
H	-2.21113766775843	2.89105299363872	-0.03251677678385
H	-2.27367322790674	4.65523389578156	-0.16509184753799
H	-1.98724515527023	3.90283246040157	1.39132439454361

4^{Ga} E = -3965.789242506538 au
ZPE = 0.08655185 au
G_{corr} = 0.05096466 au

C	2.59525083470497	2.39585916513977	-0.55481466459655
H	3.07278875945238	3.37079873271670	-0.45381550973993
C	1.26385998328988	2.32176121864453	-0.38088458560785
H	0.78900574580084	1.34484067887777	-0.48314464059363
Ga	0.16919926533028	3.91188700041940	0.04974804634878
H	0.96406055035178	5.26926106496848	0.18361797212456
Ga	3.68009573283633	0.80822177245231	-0.98243830964718
H	5.23739969280416	0.89124925880548	-1.18925500531466
H	2.91135610298935	-0.55984742149123	-1.10934995433459
C	-1.79086173031546	3.81687577548550	0.31849701464736
H	-2.20694193787444	2.86836151930703	-0.02173340734556
H	-2.29499740461766	4.63350373328403	-0.20175742936917
H	-2.01730538475245	3.92503577139011	1.38311443342842

4^{In} E = -497.79653037723 au
E = -497.21362811637 au [CASSCF(6,6)/MRACPF/def2-SVPD]
ZPE = 0.08293524 au
G_{corr} = 0.04512228 au

C	2.66650451180803	2.38590677232668	-0.56557183199572
H	3.14305638674613	3.36045875481395	-0.46439555739593
C	1.34018507603008	2.29259350460906	-0.39895911781249
H	0.86672247987260	1.31604362316321	-0.50113444584477
In	0.11679362721258	4.02546559622933	0.07313094706820

H	0.99451307353977	5.52444872991939	0.20766140633811
In	3.88164480135726	0.65734786992146	-1.03441973608292
H	5.59800228309975	0.75329389465334	-1.26702588787823
H	3.02328665648715	-0.84743576834750	-1.16561158513946
C	-2.03555309514355	3.89431267947671	0.37143805515571
H	-2.42710293635211	2.94755793092768	0.00167885112560
H	-2.53874854027341	4.71708431012874	-0.13691615036153
H	-2.25639411438431	3.97073037217779	1.43790901282344

4^{C}

E = -196.166476926203 au
ZPE = 0.13611422 au
G_{corr} = 0.10646843 au

C	-0.92622433607999	1.16320331058354	1.82955148474054
C	0.54858151585156	0.94018127140251	1.95373551514232
C	1.45161236123125	1.88622928682714	2.18806404879718
C	2.92982565545045	1.66619229876430	2.29660309736656
C	3.71896402733727	2.41575030213283	1.21723346417812
H	4.79372012561193	2.26573285251687	1.33890829617233
H	3.52389423168677	3.48988681655024	1.26361325516296
H	3.43625886709997	2.07032264439392	0.22087277284292
H	3.27547378231785	2.00031425466117	3.28174699046420
H	3.14492319160196	0.59576814369122	2.23708346262150
H	1.11460987874412	2.91653723889797	2.29627177942270
H	0.89018335616447	-0.08721817427905	1.84170726331649
H	-1.18022730879180	2.21891690466976	1.93863374839190
H	-1.29710271488446	0.82268892369563	0.85798777969269
H	-1.47687989334140	0.60035000549186	2.58960352168753

4^{Si}

E = -698.202610829344 au
ZPE = 0.11099999 au
G_{corr} = 0.07809853 au

C	0.32295677014913	1.00125292076761	2.19700278938241
C	1.26000082328486	1.50326386156051	1.38213441765215
C	3.99145973988915	2.85895378275469	1.86994937015564
H	5.06736387093329	2.70182109148210	1.97091650053005
H	3.63134086554170	3.34865452858214	2.77685808890083
H	3.82850916609916	3.54004563418376	1.03168496871400
H	0.93021261234354	2.12131727080379	0.54564936439242
H	0.64890991654683	0.38196386045185	3.03318999574780
Si	3.09785586736600	1.23211612058144	1.59114784517668
Si	-1.50901516489399	1.29384557561869	1.99372876756796
H	3.64279022135227	0.58052903261791	0.37107770673947
H	3.29514236230571	0.32089579863706	2.74674421364480
H	-1.73229486553562	2.14859042230030	0.80322121987269

H	-2.23151410305135	0.00853770377234	1.82129735575811
H	-2.06892708233079	1.96473639588567	3.19413839576480

4^{Ge} E = -4271.294106789626 au
ZPE = 0.10729533 au
G_{corr} = 0.07202566 au

C	0.31915208454999	0.86790642993448	1.88083341116253
C	1.20042622980670	1.79009736389543	2.27078885368842
C	4.10038169465719	2.68697293610638	1.15384404061804
H	5.14870956518668	2.39515692641409	1.08854131335115
H	4.04866458223081	3.70864566179322	1.53049850688800
H	3.66504421754142	2.65912675773536	0.15520107969109
H	0.84623498778609	2.78172940425606	2.54840434368907
H	0.67466084054960	-0.12293553769230	1.60449645376610
Ge	3.13142728267730	1.46680176825250	2.35241754949418
Ge	-1.60218012096611	1.20273231967805	1.76337766065886
H	3.35970478239136	-0.00199272310658	1.93623508542775
H	3.63518952276160	1.66385581486723	3.80016772265943
H	-1.85981504251633	2.66520247414970	2.17083278890377
H	-2.11187446621647	0.96754611840947	0.32664069629616
H	-2.38053783043984	0.26078198530686	2.70254115370539

4^{Sn} E = -547.356101202813 au
ZPE = 0.10057453 au
G_{corr} = 0.06308830 au

C	0.27592808432089	0.94381970379724	2.20160328988136
C	1.16715510253251	1.45899134774956	1.35724558510086
C	4.28054380689115	3.04785257712580	1.89364276626477
H	5.35404051422376	2.89551018351404	1.99635937772993
H	3.89441942215866	3.50822245884811	2.80195084546975
H	4.09540097933747	3.71347328954789	1.05129858356324
H	0.82749860813459	2.07307620864180	0.52494092048053
H	0.61100658730175	0.32812698498744	3.03393611332278
Sn	3.28905721678846	1.15754676885361	1.55936082779572
Sn	-1.84023264231170	1.26592107158240	2.01243926848965
H	3.92103966014816	0.42663434544868	0.13771441730060
H	3.51277972692326	0.10721059642684	2.89982215903405
H	-2.08554580303211	2.23921487743450	0.62404955891803
H	-2.67035100106681	-0.22386526939976	1.83573575183439
H	-2.45794926235015	2.06478885544170	3.39864153481413

4^{N} E = -228.213235750432 au
ZPE = 0.11390264 au

G_{corr} = 0.08427669 au

C	2.31078976251540	-1.59559349701486	0.04193904888572
H	2.78464893264410	-2.53202935900723	-0.25797054071923
H	2.51979412029585	-0.84674486720931	-0.73442298312859
H	1.23439428754896	-1.76402114121743	0.08227502558178
C	4.16272899351716	-1.11578408520438	1.54805361744974
C	4.79265391746986	-0.23393585266222	2.32866823258575
N	2.79225655763282	-1.21719789701331	1.36056551563291
H	2.27296648711682	-0.45535352891043	1.77416871285136
N	6.20087768056122	-0.34378088011040	2.55828119928288
H	6.66359320666076	0.55289535280162	2.46891147341736
H	6.40863494044991	-0.69573059937958	3.48662255260786
H	4.74817281371845	-1.87557033113075	1.03958825059461
H	4.22510154986862	0.55602208605829	2.82439064495782

4^{P} E = -800.683010076002 au
ZPE = 0.09710934 au
G_{corr} = 0.06484006 au

C	2.13614533678351	-1.59695296651657	-0.19210673348996
H	2.53380218104988	-2.53715483460420	-0.57679432718683
H	2.63248202005584	-0.76776065254897	-0.69471235598723
H	1.06873225272995	-1.57175143610631	-0.41564153355748
C	4.16586540674324	-1.38677562218165	1.76508218099827
C	4.83720188201465	-0.39605674676512	2.35540444236257
P	2.36087960113682	-1.56458027884245	1.65182168238194
H	2.03360462606059	-0.20344281090124	1.87919743821306
P	6.65849138162056	-0.46376185591360	2.52162466253445
H	6.93055745627716	0.88954161117744	2.18177356140424
H	6.73547638564930	-0.19014030217000	3.91510557411479
H	4.72550721950561	-2.23887466639874	1.38113323041795
H	4.29786750037282	0.46088596177142	2.74918292779421

4^{As} E = -4587.796260506876 au
ZPE = 0.09270943 au
G_{corr} = 0.05788424 au

C	2.05894478588731	-2.08096879252475	0.07712020741705
H	2.37225313527000	-3.02005490072816	0.53094648282777
H	2.68759965560308	-1.86613880715934	-0.78522516350725
H	1.02120189100356	-2.17141406176133	-0.24209289044990
C	4.13368635268772	-0.42138247346344	1.38495271284779
C	4.87571380467265	-0.39553431114162	2.48560001558898
As	2.18437880546027	-0.64753014886173	1.45291022057271
H	1.89254674887159	0.48810556126930	0.46780894819443
As	6.82870117409959	-0.19711310143978	2.39123977930654

H	6.91647130873706	0.86807477691748	3.48538718700317
H	7.13172634961496	-1.30423053613600	3.40237595931661
H	4.59980222290724	-0.33575778197869	0.40577632640344
H	4.41358701518481	-0.48288002299190	3.46427096447862

4^{Sb} E = -598.045430198830 au
ZPE = 0.08781515 au
G_{corr} = 0.05096437 au

C	1.93726886984792	-2.17963093420002	-0.03223322842430
H	2.25624803582710	-3.13090420679963	0.39041647546591
H	2.59633290954262	-1.90264035106770	-0.85265691137345
H	0.91605414661372	-2.27326006314184	-0.39889231518930
C	4.14711149400129	-0.40006073467221	1.43556090606406
C	4.91662817635715	-0.37174502577064	2.51693594860222
Sb	2.00125493329062	-0.66045636443921	1.53567147048447
H	1.69114153042358	0.61985848025948	0.42640071606698
Sb	7.06532600307213	-0.14685801087848	2.38590137966362
H	7.13714114631037	1.03811659072107	3.62914244787069
H	7.38357676260675	-1.38808585833398	3.53210784611971
H	4.58299097444034	-0.31064647075589	0.44235043359674
H	4.48553826766629	-0.46051165092094	3.51036558105259

4^{Bi} E = -546.751489822818 au
ZPE = 0.08516710 au
G_{corr} = 0.04655348 au

C	1.90653384019282	-2.24221605696907	-0.07059112821691
H	2.24339265330919	-3.17888069392633	0.36903783596833
H	2.58001345514009	-1.93768976996445	-0.86799561893083
H	0.89551549775534	-2.35500346191012	-0.45752822030063
C	4.14679058562603	-0.34930807365456	1.45118995805369
C	4.91745148808471	-0.35175804513618	2.52671697242758
Bi	1.90243151501270	-0.64716331154491	1.56483286701138
H	1.58065516587664	0.66591018097425	0.36581831576433
Bi	7.16672499322953	-0.10393062745208	2.37917136830280
H	7.22291842854586	1.07756811502420	3.73986310402149
H	7.48457351627952	-1.44520573724056	3.54190992484053
H	4.56068883497264	-0.22712578125140	0.45253900372015
H	4.50892327597485	-0.47202133694876	3.52610636733806

4° E = -267.917368251515 au
E = -267.450002026755 au [CASSCF(6,6)/MRACPF/def2-SVPD]
ZPE = 0.08864020 au

G_{corr} = 0.05934650 au

C	2.35140005987719	-1.76062920617269	0.23069054619969
H	2.55938198986823	-2.75310109902425	0.64434132329212
H	2.82233227511235	-1.68048460948870	-0.75577928292178
H	1.27627344189414	-1.62739777815808	0.12682021745376
C	4.17191732092752	-0.69632266983165	1.24166125782822
C	4.72606266981954	-0.38814574841985	2.40789333418975
H	4.76809832953720	-0.86012600047793	0.34719754589219
H	4.12202663673914	-0.23185332941076	3.29521169542111
O	2.81170947372538	-0.73685027345932	1.09936612822572
O	6.07956831674270	-0.16367503057151	2.50823231252257
H	6.35858231575646	-0.31600578498519	3.41599324189658

4^{S}

E = -913.145660265064 au
ZPE = 0.08009133 au
G_{corr} = 0.04842645 au

C	2.16410545472186	-1.93716849069759	0.05314492235661
H	2.44116067150247	-2.88672870708199	0.50799134654264
H	2.74112825753484	-1.77643204507909	-0.85756559749720
H	1.10458958771117	-1.95080769855183	-0.19857000044857
C	4.15540755907250	-0.56153437987582	1.33687858590857
C	4.79052706492119	-0.27371404736891	2.47336971243059
H	4.70837350667137	-0.72245913402316	0.41567291874584
H	4.24361222895747	-0.13252526504799	3.39808625204221
S	2.40743578057336	-0.55934893986161	1.21226055783953
S	6.52087790767496	0.06194122570304	2.50680475942824
H	6.77013481065858	-0.47581404811499	3.71355486265147

4^{Se}

E = -4917.917324727201 au
ZPE = 0.07718083 au
G_{corr} = 0.04286649 au

C	2.09599739992741	-2.00729254984971	-0.00546310429631
H	2.42715688139384	-2.92039273869830	0.48184887449526
H	2.67917359083298	-1.81702006754546	-0.90389299940780
H	1.04231884509765	-2.08435125792060	-0.26547278095691
C	4.16431364941413	-0.48305789066980	1.35087944262015
C	4.80191405766543	-0.22650994500776	2.48712907002027
H	4.69700050856483	-0.63036529697409	0.41728314306174
H	4.27745536777662	-0.09678380309068	3.42534239234839
Se	2.26115893732439	-0.48356661246740	1.22574826449844
Se	6.68954686599631	0.09941774202407	2.52568301625241
H	6.91131672600633	-0.56466910980024	3.82254300136434

4^{Te} E = -652.555044644053 au
ZPE = 0.07447500 au
G_{corr} = 0.03820696 au

C	2.00602903162979	-2.10101716517926	-0.10446189232992
H	2.36984797664851	-3.00182790006321	0.38065064886535
H	2.61661460290794	-1.85006921690832	-0.96796223122777
H	0.97064686751033	-2.23123946776693	-0.41191364162474
C	4.16000436188578	-0.39698108479092	1.41029273619685
C	4.81558994044543	-0.15266862358043	2.53721255005132
H	4.67364838031092	-0.51754504989464	0.46215060579874
H	4.31349756657305	-0.04609199923636	3.49108385676108
Te	2.05150675528212	-0.45258947514146	1.29469835765472
Te	6.90072644916751	0.22925269751766	2.55460141480667
H	7.16924089763843	-0.69381424495608	3.91527591504763

4^{Po} E = -591.980337213346 au
ZPE = 0.07296514 au
G_{corr} = 0.03507742 au

C	1.97176672115646	-2.17556445071810	-0.11911409868914
H	2.31904263540397	-3.03141332766225	0.45025733212618
H	2.61885391865160	-1.97375313243795	-0.96759808879129
H	0.94726743494562	-2.32366773079726	-0.45248347636131
C	4.18197870026109	-0.30778225438555	1.38960652205004
C	4.80341805802959	-0.09620129203955	2.53593501919762
H	4.70559214937934	-0.41253581489592	0.44584933347676
H	4.29256265497082	-0.00684068270555	3.48635298216253
Po	1.96433663277785	-0.35202009322044	1.22630845611133
Po	7.00113686371114	0.28207427717279	2.60959484614214
H	7.24139706071245	-0.81688702831022	3.95691949257512

5^{N} E = -212.199697514936 au
ZPE = 0.12433933 au
G_{corr} = 0.09490241 au

N	-1.15884857885029	2.93019820199749	1.18584834897513
C	-0.10251503357953	3.38174346464580	1.70661948016245
H	0.29630249311063	4.36831983303560	1.41955104154059
C	-1.83588921134814	3.73187468182767	0.18290357085261
C	0.67445649015156	2.62764454124056	2.73584423529282
C	-1.90979312901684	2.98049773912819	-1.14228311894933
H	1.70335519123754	2.47185281209126	2.39831639150085
H	0.20816584258406	1.66393635039750	2.93352152534934
H	0.73087642845492	3.20043343513417	3.66644365848609

H	-2.85047997656629	3.92347475349185	0.54508451520680
H	-1.34570183939997	4.70746504950582	0.03991383127450
H	-0.90919185588935	2.80492720430720	-1.54222159426100
H	-2.47795158943738	3.55330196417465	-1.87789763265972
H	-2.39357389145092	2.01258228902219	-1.00478579277114

5[P] E = -498.426152591566 au
E = -498.007055049443au [CASSCF(8,6)/MRACPF/def2-SVPD]
ZPE = 0.11974061 au
G_{corr} = 0.08888762 au

P	-1.40345739172913	2.48272498138103	1.36659296292381
C	-0.00879639181369	3.24734290797214	1.86434231271781
H	0.26784946780256	4.21589082636411	1.45223207129699
C	-2.10046949739683	3.65833492986998	0.09709983053380
C	0.93213444708293	2.68676684204942	2.88397239495865
C	-1.98852330740698	3.10212499389400	-1.32604319766477
H	1.93496762609263	2.56414410515564	2.46215259056479
H	0.59818457074532	1.71753172098474	3.25755283870269
H	1.03164613389313	3.36636730849608	3.73658104971521
H	-3.15362233279314	3.81408227974375	0.34873790445870
H	-1.60251555511708	4.62691033662949	0.17616723234592
H	-0.94344288909068	2.99633117536817	-1.62258544687804
H	-2.47991692307165	3.76227227005076	-2.04445909351332
H	-2.45482661719736	2.11742764204063	-1.40548499016228

5[As] E = -2391.981967604694 au
E = -2391.39870522949au [CASSCF(8,6)/MRACPF/def2-SVPD]
ZPE = 0.11844104 au
G_{corr} = 0.08633039 au

As	-1.53414710571457	2.39661997631926	1.47138332774651
C	0.00269955287221	3.20820096454497	1.89750937709431
H	0.26765190816094	4.14423625586866	1.41145483117570
C	-2.20699076633540	3.65660430866259	0.07235852183160
C	0.98195668737550	2.69300861344916	2.89934930237042
C	-1.95571069797605	3.13891402721391	-1.34318626851277
H	1.96132089896435	2.52617676587627	2.43710068595306
H	0.65607331041300	1.75396432024470	3.34988407968163
H	1.13936863308572	3.42027321993051	3.70349341396558
H	-3.27768271026608	3.77876723145824	0.24896964854400
H	-1.73382408666280	4.62698410586363	0.22834292009336
H	-0.88642202776641	3.06028596316494	-1.54868631438925
H	-2.39361703373137	3.80640229543795	-2.09045321125592
H	-2.39146522241900	2.14781427196506	-1.49066185429828

5^{Sb} E = -397.103853464947 au
E = -396.61232005295 au [CASSCF(8,6)/MRACPF/def2-SVPD]
ZPE = 0.11708901 au
G_{corr} = 0.08383649 au

Sb	-1.63031569225944	2.30841042493718	1.51558043838008
C	0.10171285247223	3.15644819236852	2.01161943053427
H	0.40231169101298	4.05712884755740	1.48099752686797
C	-2.16336896534893	3.73634523483737	-0.06797329461860
C	1.04900310167237	2.68495461050153	3.06231701956495
C	-2.11737831311683	3.12409388636181	-1.46609153750649
H	2.03451448026085	2.46838411183705	2.63297777246431
H	0.69965992654906	1.78476970153866	3.57121731279385
H	1.21015752790573	3.45990194231134	3.82124111856517
H	-3.16518631016845	4.10505260028158	0.16061588120926
H	-1.47405960117384	4.57695554098839	0.02239684977265
H	-1.11181778262061	2.78049002629041	-1.71757279195098
H	-2.41864512307852	3.85022602830748	-2.22730326746912
H	-2.78737645210659	2.26509117188116	-1.55316399860733

5^{Bi} E = -371.458104024735 au
E = -370.900557299804 au [CASSCF(8,6)/MRACPF/def2-SVPD]
ZPE = 0.11621401 au
G_{corr} = 0.08325827 au

Bi	-0.65910442145706	1.94202547837346	0.73225083840425
C	0.17062314380460	3.17170407882220	2.19411507435826
H	-0.06402296438366	4.23318763153975	2.15797144365861
C	-1.78675833811624	3.63664463131527	-0.34738651325541
C	1.07331684369755	2.75047987945073	3.30274632094188
C	-2.49137482873439	3.14938845705299	-1.60635214020241
H	2.03246031780301	3.28093949729139	3.25185300331282
H	1.28308951112930	1.67917014842401	3.29493713609141
H	0.64085681740886	3.00077307409702	4.27957986876955
H	-2.49103007926583	4.04572284303231	0.37684113290116
H	-1.04326915589885	4.40174213896663	-0.57030278002021
H	-1.78476539855540	2.73320219946279	-2.32882803200335
H	-3.02586399992664	3.96272811181770	-2.10748349801476
H	-3.22494610750522	2.37054415035372	-1.38308339494181

6^{N} E = -226.997002798904 au
ZPE = 0.09031936 au
G_{corr} = 0.06167945 au

C	-0.09034993736075	3.04254177503205	0.84154232805479

H	0.64305819635239	3.09436828120705	0.02689524637467
H	-0.88973790519569	2.31000734968770	0.74681254433739
N	-0.04839931477882	3.78043753998185	1.86181011332664
C	1.02895289705884	4.75017085673558	1.95819853552300
H	1.63537773148274	4.47562396908427	2.83071722239934
C	0.46741792709525	6.12286229473816	2.20891875537292
H	1.68223640438844	4.77933786802135	1.07765456284693
H	-0.32222425532412	6.16486564182872	2.96747855032022
N	0.89922402285686	7.12915148798636	1.57694763689230
H	0.42987074342484	7.98393706569675	1.87715713455174

6^P E = -799.451971356681 au
E = -798.965750423657 au [CASSCF(8,6)/MRACPF/def2-SVPD]
ZPE = 0.07928486 au
G_{corr} = 0.04838771 au

C	0.21512534877962	2.93182463816145	1.91296727745126
H	0.73064021579072	3.47590972272179	2.69597066519163
H	-0.01114638869869	1.89190634874098	2.11977203242015
P	-0.23876517897360	3.55580427776955	0.44909172943533
C	0.37795967410288	5.35013558445312	0.47792495247514
H	1.33622146950601	5.34723304384804	-0.05031872852581
C	0.48370397865449	5.96431423230516	1.81979833793339
H	-0.33448161271440	5.89365052663449	-0.14953685944545
H	-0.46265478852143	6.17136332326391	2.30937149784408
P	1.94174415291011	6.26975590177931	2.58780990528749
H	1.39707963916425	6.78140653032202	3.80128181993273

6^As E = -4586.565241155862 au
E = -4585.74910062949 au [CASSCF(8,6)/MRACPF/def2-SVPD]
ZPE = 0.07656394 au
G_{corr} = 0.04356311 au

C	-0.48977587096790	3.40963635592888	0.60215701191260
H	-0.50832117048470	4.42670544382868	0.22716781002122
H	-1.09743504108086	2.68314034574141	0.07630470116870
As	0.45746972699739	2.87565826169553	2.00441673442861
C	1.42355237727087	4.59730401599975	2.55038258458709
H	1.53554520830913	4.48215420581344	3.63049326635968
C	0.70775378632684	5.82286463829334	2.19143733944689
H	2.41033850755080	4.52247279018235	2.08928059701558
H	-0.17352788230204	6.04840686900595	2.78205197510512
As	1.16929523619491	6.89571868332941	0.81795689615977
H	0.00053163218554	7.86924252018105	1.00248371379471

6^Sb E = -596.812096227292 au
E = -596.177937594001 au [CASSCF(8,6)/MRACPF/def2-SVPD]
ZPE = 0.07323969 au
G_{corr} = 0.03818450 au

C	-0.10864325128923	2.74418128949312	0.33199075402562
H	0.93986159236611	2.83071895684628	0.07138719365916
H	-0.70790513543284	2.13068531613853	-0.33059993108947
Sb	-0.93455079128428	3.65998399645862	1.87457975568322
C	0.93130746762108	4.72363469796342	2.54357769252646
H	1.09260282136926	4.40816169793647	3.57512655920090
C	0.68315985675020	6.15305489810935	2.40054434738884
H	1.71092313922197	4.32370481962286	1.89738889035716
H	0.10318183697768	6.61049083016398	3.19678765583802
Sb	1.24495512052909	7.33202162835846	0.86793580088605
H	0.48053385317092	8.71666599890875	1.54541391152397

6^Bi E = -545.526259214943 au
E = -544.760601011592 au [CASSCF(8,6)/MRACPF/def2-SVPD]
ZPE = 0.07167427 au
G_{corr} = 0.03521477 au

C	-0.62349696493552	2.67794386906156	1.02324918915277
H	-0.36888464959439	2.45987264499992	2.05398129747004
H	-1.47153315799460	2.13549675636651	0.62122296633924
Bi	0.32975178570773	4.13933773063050	-0.08709568563103
C	1.73783700334911	4.93482169611554	1.65181977962114
H	1.89828436365118	4.06653853294078	2.28811751087167
C	0.88784166103138	5.97063787444262	2.16603887947850
H	2.64650649480649	5.24730751720609	1.14281723344812
H	0.13088186716576	5.65008658656776	2.87514090835205
Bi	0.84696656582975	8.02038631139958	1.59125781815739
H	-0.57872845901690	8.33087461026886	2.64758273274004

7^N E = -228.222714374713 au
ZPE = 0.11384147 au
G_{corr} = 0.08453989 au

N	0.85995205927219	4.89088492365496	1.60520022696568
C	0.02103354224224	4.81463031932821	0.42715677941498
C	1.08109207104023	3.83289536836141	2.25496973731016
H	0.67858995408364	2.85627526483824	1.94665851600420
H	0.58198979054593	5.18262617215827	-0.43510952340185
H	-0.34318737507007	3.80066676312869	0.21086046175581
H	-0.83611170427496	5.47994225017194	0.55544868168576
C	1.93585586812388	3.81664568282726	3.48800665801103
H	1.30968804084336	3.51458939515657	4.33451737787424

H	2.28830072867684	4.83624769128814	3.68086481083038
N	2.99077343450105	2.81041987898270	3.33928907437083
H	3.68617392671568	3.12395571256200	2.67196463446389
H	3.46538120329998	2.65041329754157	4.21903588471488

7^P E = -800.684668537831 au
E = -800.188504507727 au [CASSCF(8,6)/MRACPF/def2-SVPD]
ZPE = 0.09940537 au
G_{corr} = 0.06728726 au

P	1.00886086080009	5.30004505924936	1.51453149738778
C	-0.16001166432067	4.88590341663796	0.13387186801948
C	1.02918959918707	3.90407956625795	2.42582520545738
H	0.44074602073614	3.03532147621727	2.13833477528490
H	0.36616720000283	4.96182188495305	-0.82033116467408
H	-0.58162241126290	3.88528110752482	0.22946065821937
H	-0.97186951194534	5.61612538051150	0.11465499572482
C	1.86999555321815	3.73727276113747	3.64680777315235
H	1.26066242042664	3.37663535088584	4.48181139013442
H	2.33376883283734	4.67686534079828	3.94619498593042
P	3.17293404358438	2.40923875561113	3.35344233286575
H	4.04975398225038	3.20933147642396	2.57731846608639
H	3.90095661448586	2.61227114379134	4.55694053641096

7^As E = -4587.798791597225 au
E = -4586.97137920671 au [CASSCF(8,6)/MRACPF/def2-SVPD]
ZPE = 0.09593211 au
G_{corr} = 0.06157050 au

As	1.08652691632348	5.41208867389936	1.46106442430467
C	-0.22451966665680	4.90400125868084	0.05495686863994
C	1.02223553139698	3.94298427368471	2.48726333816213
H	0.36525985235477	3.12123011571482	2.21324352085526
H	0.27526591359549	4.89464362980316	-0.91420278799228
H	-0.64985100448779	3.92157369194491	0.25408301973445
H	-1.02182570975897	5.64682747398127	0.02047890058165
C	1.85245513872776	3.73727823723534	3.69228173385258
H	1.26289380320376	3.35164324905747	4.52789776118774
H	2.36881133235086	4.64090353487430	4.00994110950018
As	3.22764105921533	2.29541670570305	3.32553777175192
H	4.15056793210374	3.22284320656907	2.54082752029000
H	4.00407044163131	2.51875866885156	4.62549013913170

7^Sb E = -598.047877661166 au
E = -597.404616975115 au [CASSCF(8,6)/MRACPF/def2-SVPD]

ZPE = 0.09196320 au
G_{corr} = 0.05583068 au

Sb	1.20455593622729	5.56827168347039	1.34161348907941
C	-0.32870192116347	4.93285109617805	-0.08220968952151
C	1.02048768059874	3.99601975123893	2.56971499713445
H	0.28206682547429	3.23989942104820	2.31229629360913
H	0.12350962341873	4.77057236300677	-1.05996413626270
H	-0.78793016627934	4.00887824504468	0.26618113996599
H	-1.08957006398336	5.70762821179974	-0.17030371593602
C	1.81356688339252	3.72413032661967	3.76932506903393
H	1.23472241358410	3.27750100176661	4.58001183525603
H	2.37401547839421	4.57761870149649	4.14393940493740
Sb	3.34439467384738	2.16698749385451	3.26952975143265
H	4.37784200118534	3.34295150098762	2.57166615279497
H	4.15057217530353	2.29688292348820	4.78706272847618

7[Bi] E = -546.758851555229 au
E = -545.98255256769 au [CASSCF(8,6)/MRACPF/def2-SVPD]
ZPE = 0.08988058 au
G_{corr} = 0.05207435 au

Bi	1.27353687040082	5.62985378264339	1.25923459355753
C	-0.37347068728144	4.90725792796469	-0.15425669368269
C	1.01514777813322	4.03226053648570	2.60728350501908
H	0.24021297219940	3.31020938857948	2.35916069875965
H	0.06802482153823	4.61288907646826	-1.10476044685755
H	-0.87467007439735	4.05664194414595	0.30402365874616
H	-1.08637892354843	5.71384485449079	-0.31717173229725
C	1.77644070285595	3.75700665409454	3.80604979957603
H	1.22124590748458	3.26449313781356	4.60476417761432
H	2.36950180187633	4.57960554713385	4.19562317631260
Bi	3.40228291928919	2.14210386612842	3.22037685551611
H	4.49428191015795	3.45858672987672	2.66836051509286
H	4.19337554129154	2.14543927417455	4.85017521264309

CH_2=CH_2 E = -78.439536875881 au
E = -78.305274101452 au [CASSCF(6,6)/MRACPF/def2-SVPD]
ZPE = 0.05091817 au
G_{corr} = 0.02939867 au

C	0.38523242958429	0.94509694078870	0.38675586386309
H	0.89860409377577	-0.00915281091743	0.38675592468037
H	-0.69782480959164	0.91192685604553	0.38675586734631
C	1.04758128189125	2.09262210823183	0.38675592535617
H	0.53420954339267	3.04687176115926	0.38675590804281
H	2.13063844094765	2.12579220469212	0.38675585071126

CH_3-BH_2 E = -65.797576779684 au
ZPE = 0.05549932 au
G_{corr} = 0.03220895 au

B	0.24772150350817	3.73079688623850	0.09956930597696
H	0.85840750406510	4.64805432609368	0.56139037247035
C	-1.30314861682466	3.74850246861919	0.05074439272838
H	-1.74752623308435	3.10904320550302	-0.71393647300000
H	-1.75300272124268	4.74225964203922	0.01817247625425
H	-1.60735957106632	3.31298571338038	1.01905224358734
H	0.86223000464473	2.77401925812600	-0.26727144801728

CH_3-AlH_2 E = -283.017000756422 au
ZPE = 0.04766598 au
G_{corr} = 0.02328039 au

Al	0.42447906641152	3.73395316189061	0.10775017006585
H	1.23998766042757	5.01174988719238	0.57078942885238
C	-1.53092920531067	3.73458290976206	0.09451138395267
H	-1.93277631409244	3.20011443559870	-0.77067573824959
H	-1.95841048386485	4.73935104124636	0.10855335253554
H	-1.90738398513882	3.21142992195111	0.98170477979968
H	1.22235513156769	2.43448014235874	-0.32491250695652

CH_3-GaH_2 E = -1964.481098256562 au
ZPE = 0.04782524 au
G_{corr} = 0.02229129 au

Ga	0.43327100286045	3.73367458497086	0.10820756389474
H	1.24282582998805	4.99906962776074	0.58460658682466
C	-1.54644377943444	3.73450350438509	0.09262734138726
H	-1.93462592738066	3.18470591550224	-0.76615573315064
H	-1.95878702690968	4.74328967951854	0.09256107897412
H	-1.90542523154712	3.22445009794748	0.99208654496396
H	1.22650700242341	2.44596808991501	-0.33621251289410

CH_3-InH_2 E = -230.487592778386 au
E = -230.221280945481 au [CASSCF(6,6)/MRACPF/def2-SVPD]
ZPE = 0.04605745au
G_{corr} = 0.01948236 au

In	0.51816779638548	3.73249479188988	0.11085648460679
H	1.40420011377253	5.13541621545338	0.62646921647012

C	-1.65630281600566	3.73100452561218	0.09671903511642
H	-2.03113313314471	3.18983552270962	-0.77192451319800
H	-2.05292447575194	4.74512926368479	0.09490941529426
H	-2.01511660836465	3.21972341314465	0.99273807421109
H	1.39043099310896	2.31205776750544	-0.38204684250068

CH_3-CH_3 E = -79.675371884486 au
E = -79.536219431848 au [CASSCF(8,6)/MRACPF/def2-SVPD]
ZPE = 0.07437551 au
G_{corr} = 0.05125541 au

C	-0.66800282983779	0.65385739818262	-1.31293515142985
H	-0.12282684166028	-0.27678413351344	-1.14173215316382
H	-0.55067627154633	1.27347336364917	-0.42128124917978
H	-1.72718307275635	0.40379060809260	-1.40360307458524
C	-0.15898983752621	1.37350198380431	-2.55933104498186
H	0.90019065330116	1.62356770884469	-2.46866302366946
H	-0.70416497533903	2.30414428459042	-2.73053304115872
H	-0.27631710463517	0.75388669634962	-3.45098530183126

CH_3-SiH_3 E = -330.690172279490 au
ZPE = 0.06081520 au
G_{corr} = 0.03593388 au

C	3.77114207905829	2.62708220314037	1.50494688491184
H	4.83403184334689	2.37803537210273	1.50514620049210
H	3.62408147608335	3.48082674978087	2.16926743051235
H	3.49599830687446	2.93401125509424	0.49397081271463
Si	2.73958600407415	1.16131301568059	2.06190521339391
H	3.11449364507584	0.75101257515020	3.43794260728857
H	1.29607587554873	1.50589729864500	2.05555051781140
H	2.94610940993830	0.00312286040601	1.15736066287519

CH_3-GeH_3 E = -2117.237360801692 au
ZPE = 0.05913599 au
G_{corr} = 0.03286617 au

C	3.80105161999802	2.66971003883639	1.48883315298583
H	4.85874102294007	2.40736143216998	1.48883052521510
H	3.64981918141237	3.51387367521740	2.16121172373087
H	3.51446291471109	2.96903969465583	0.48083037585986
Ge	2.72034124873572	1.13420025202827	2.07255252588273
H	3.11632465781405	0.71136660207008	3.50050855741028
H	1.22299072534090	1.49883429647955	2.06637033662969
H	2.93778726904777	-0.06308466145751	1.12695313228564

CH_3-SnH_3 E = -255.269760743869 au
ZPE = 0.05556147 au
G_{corr} = 0.02805300 au

C	3.86647299779083	2.76251644347787	1.45351387131845
H	4.92082489868521	2.49061971549908	1.44434648878016
H	3.71944499510148	3.59792203779644	2.13634459548760
H	3.56680634338509	3.06406026136731	0.45090649947832
Sn	2.68017083402188	1.07648269962670	2.09427928144369
H	3.13453155151171	0.60366630235454	3.67837502965858
H	1.01706380997119	1.49381043190689	2.09715865979536
H	2.91620320953260	-0.24777656202884	1.03116590403782

CH_3-NH_2 E = -95.699472477154 au
ZPE = 0.06370738 au
G_{corr} = 0.04076742 au

C	2.43379750094523	0.64409609268184	0.23803396473924
H	2.80533188950474	-0.38198336451734	0.25997331128764
H	2.80421078843273	1.14073718852884	1.13653866510437
H	2.87920077348072	1.14547010801241	-0.63268878821707
N	0.97118869323675	0.62787243222819	0.26548882941925
H	0.60185930768921	1.57117289612663	0.26328404965758
H	0.60209040671062	0.16545028693943	-0.55688238199102

CH_3-PH_2 E = -381.932156838862 au
E = -381.714227983371 au [CASSCF(8,6)/MRACPF/def2-SVPD]
ZPE = 0.05420142 au
G_{corr} = 0.02965884 au

C	2.58881259145355	0.61519785672567	0.28867448859830
H	3.00342690370111	-0.39355173486672	0.27274807132142
H	3.00370562821839	1.12966682566147	1.15633371496337
H	2.89520227749822	1.13927441408462	-0.61490490073638
P	0.74135300025047	0.49899434849039	0.49167130793615
H	0.43273605980151	1.85486942753282	0.20484755525085
H	0.43244289907675	0.06836450237175	-0.82562258733371

CH_3-AsH_2 E = -2275.489147056667 au
E = -2275.06458586079 au [CASSCF(8,6)/MRACPF/def2-SVPD]
ZPE = 0.05202927 au
G_{corr} = 0.02608054 au

C	2.64391941817136	0.60773106083848	0.30066629573056
H	3.05794554111136	-0.39938455842924	0.27656169535500
H	3.05619817613806	1.13281922241490	1.16124965327053
H	2.91135114839662	1.13488622070857	-0.61138144202228
As	0.67111136516075	0.47048751647441	0.53821854804925
H	0.37896723915618	1.93281938006909	0.20483932947979
H	0.37818647186567	0.03345679792378	-0.89640642986285

CH_3-SbH_2 E = -280.613984311756 au
E = -280.321286856143 au [CASSCF(8,6)/MRACPF/def2-SVPD]
ZPE = 0.04930404 au
G_{corr} = 0.02224246 au

C	2.72807438192823	0.59618392504005	0.32009134624176
H	3.15254578929629	-0.40580021833114	0.28473612718519
H	3.14958373696100	1.13163483938593	1.16931729585179
H	2.95598110849643	1.12677118476467	-0.60045955152494
Sb	0.56860216281261	0.43108240685660	0.60438244886358
H	0.27195335394704	2.07735567587287	0.21464752122275
H	0.27093882655839	-0.04441217358899	-1.01896753784013

CH_3-BiH_2 E = -254.967299524267 au
E = -254.60740753351 au [CASSCF(8,6)/MRACPF/def2-SVPD]
ZPE = 0.04792955 au
G_{corr} = 0.01988696 au

C	2.77040880366939	0.59133511859799	0.32808400798678
H	3.19114064994861	-0.41135343828635	0.28860332523156
H	3.18725926857842	1.13370298934074	1.17437306836361
H	2.97251894650703	1.12276697725488	-0.59705213391158
Bi	0.51480163321680	0.41034195198583	0.63871768541053
H	0.23110639329805	2.13783327223713	0.21438122473705
H	0.23044366478170	-0.07181123113023	-1.07335952781795

CH_3-OH E = -115.555028214015 au
E = -115.351595143124 au [CASSCF(6,6)/MRACPF/def2-SVPD]
ZPE = 0.05097328 au
G_{corr} = 0.02818610 au

C	2.42968742338976	0.65297044185859	0.25571722243951
H	2.76917689352244	-0.38209496937041	0.25548404582483
H	2.83206547117104	1.14770045724797	1.14705878485951
H	2.83163706400850	1.14797540698902	-0.63566921401902
O	1.00881707570995	0.61996891789204	0.25604185950158
H	0.67902402219830	1.52396111538279	0.25683218139360

CH_3-SH E = -438.164950352546 au
ZPE = 0.04581850 au
G_{corr} = 0.02157168 au

C	2.55992818472709	0.61390850242356	0.25565603106621
H	2.94514026061403	-0.40461950650791	0.25524803111189
H	2.91605166371816	1.12305714514653	1.14898743560980
H	2.91565552802799	1.12364281494138	-0.63750090736517
S	0.74187748959690	0.46948758080854	0.25603011358332
H	0.47175482331583	1.78500483318791	0.25704417599395

CH_3-SeH E = -2440.551335825452 au
ZPE = 0.04430450 au
G_{corr} = 0.01860218 au

C	2.61449750710168	0.59895130964493	0.25561789612065
H	3.00475511799872	-0.41638122892192	0.25520437728585
H	2.94649215398754	1.11650108967612	1.15101490103680
H	2.94617695726819	1.11713398695346	-0.63953071227265
Se	0.65122137037194	0.42294898424360	0.25592518349317
H	0.38726484327192	1.87132722840380	0.25723323433617

CH_3-TeH E = -307.870051773440 au
ZPE = 0.04267259 au
G_{corr} = 0.01591950 au

C	2.68301323919731	0.57760596305847	0.25557023311709
H	3.09103056257911	-0.43063143445106	0.25512858161159
H	2.99608540688926	1.10364671030088	1.15187931085959
H	2.99585659250005	1.10436842246619	-0.64039474340664
Te	0.52747470892939	0.35694268156136	0.25580840117784
H	0.25694743990486	1.99854902706416	0.25747309664052

CH_3-PoH E = -277.582800109094 au
ZPE = 0.04182257 au
G_{corr} = 0.01417807 au

C	2.72138210279590	0.56851513152929	0.25554530737542
H	3.12841540371144	-0.43968233102658	0.25511082309875
H	3.01836536909772	1.09911004080916	1.15365980077730
H	3.01819286461821	1.09984798653863	-0.64218927653566
Po	0.46523062032671	0.32514137013763	0.25572758608125
H	0.19882158945001	2.05754917201187	0.25761063920293

CH_2=NH E = -94.474595711883 au
ZPE = 0.03975471 au
G_{corr} = 0.01784885 au

N	-0.91252667540294	0.52302465462313	0.52009968359698
H	-1.81157186474426	0.09479082380177	0.74904932707249
C	-0.21124103148818	-0.23890150308353	-0.20359156678058
H	0.77643350465744	0.09754412545411	-0.52095079691550
H	-0.51725193302206	-1.23264358079547	-0.54747120697339

CH_2=PH E = -380.698401953028 au
E = -380.488185044388 au [CASSCF(8,6)/MRACPF/def2-SVPD]
ZPE = 0.03350368 au
G_{corr} = 0.01004203 au

P	-0.93060225036893	0.79288524708493	0.66338450455867
H	-2.10712136642879	0.00722676092153	0.85063335676530
C	-0.08991662573356	-0.28262314190945	-0.28526766792258
H	0.90042073365653	-0.01692108837998	-0.63878046584687
H	-0.44893849112525	-1.25675325771701	-0.59283428755453

CH_2=AsH E = -2274.253966816173 au
E = -2273.87870951549 au [CASSCF(8,6)/MRACPF/def2-SVPD]
ZPE = 0.03205370 au
G_{corr} = 0.00727674 au

As	-0.94064290979094	0.86375481571746	0.70356262913468
H	-2.19240017614208	-0.00543921063307	0.88630377251486
C	-0.05181066481508	-0.29988694017356	-0.31293293635698
H	0.93845007414007	-0.04509138454516	-0.67131376126694
H	-0.42975432339197	-1.26952276036566	-0.60848426402562

CH_2=SbH E = -279.377645139849 au
E = -279.093031210702 au [CASSCF(8,6)/MRACPF/def2-SVPD]
ZPE = 0.03026112 au
G_{corr} = 0.00451724 au

C	-1.62309850799709	0.47320203637855	-0.00009300625875
H	-0.79252777718015	-0.22054797475481	-0.00632368001462
H	-2.60962140691398	0.02497602263871	0.00265699038677
Sb	-1.41649231932091	2.44266620511778	0.00586757021542
H	0.30598398141215	2.39775722061976	-0.00210787432882

CH_2=BiH E = -253.733656015053 au
E = -253.380309562906 au [CASSCF(8,6)/MRACPF/def2-SVPD]
ZPE = 0.02936923 au
G_{corr} = 0.00273683 au

Bi	-0.96496988639926	1.03250636999861	0.79994554386096
H	-2.41533804057838	-0.02650898950136	0.98507280946595
C	0.03990367573952	-0.33963256783928	-0.37854950038134
H	1.03237804489324	-0.11929253807232	-0.75445997841095
H	-0.36813179365512	-1.30325775458563	-0.65487343453462

CH_2=C=O E = -152.360972698277 au
ZPE = 0.03152568 au
G_{corr} = 0.00793395 au

C	-0.49316902650322	0.52525985133606	-0.00000012014362
H	0.05194409989926	-0.40605082950677	0.00000005697985
H	-1.57227740885125	0.53195073531458	0.00000005670493
C	0.16003722510022	1.65930245808630	-0.00000003667546
O	0.73739971035500	2.66495899476982	0.00000004313430

(1^O→ CH_2=C=O)‡ E = -152.227655826773 au
ZPE = 0.02761524 au
G_{corr} = 0.00369023 au
ν= -514.75 cm^{-3}

C	-0.76532062061216	-0.44116885291061	0.45412511862863
H	1.25718021284142	-0.49405244678379	-0.12194681475370
H	-1.43714433676432	-0.72669830691020	-0.36712661909199
C	0.40873445257940	0.22370058776709	0.04623456599838
O	0.53655029195567	1.43821901883748	-0.01128625078132
